# Study on the Preparation of a Photo-Responsive Hydrogel Loaded with Berberine–Asiaticoside Cocrystal and Its Therapeutic Effect on Infected Wounds

**DOI:** 10.3390/gels12070620

**Published:** 2026-07-09

**Authors:** Muxi Sui, Jin Niu, Shuwen Pang, Shuang Zhao, Pingxi Zhou, Mengdi Zhao, Yongai Xiong, Jing Li

**Affiliations:** 1 First Clinical College, Liaoning University of Traditional Chinese Medicine, Shenyang 110033, China; mxsui0520@163.com (M.S.); shuwen_pang@163.com (S.P.); zs13804052740@163.com (S.Z.); zhoupingxi0820@163.com (P.Z.); zhaomengdimhz@163.com (M.Z.); 2 School of Medical Humanities, China Medical University, Shenyang 110122, China; 2023140067@cmu.edu.cn; 3 Key Laboratory of Basic Pharmacology of Ministry of Education and Joint International Research Laboratory of Ethnomedicine of Ministry of Education, Zunyi Medical University, Zunyi 563000, China

**Keywords:** infectious wounds, berberine, asiaticoside, cocrystal, photodynamic

## Abstract

Infectious wounds are plagued by persistent infection, uncontrolled inflammation, and delayed repair, while traditional therapies suffer from the poor solubility of natural drugs, low bioavailability, and bacterial drug resistance. To address these issues, this study developed a photo-responsive chitosan composite hydrogel (BBR-AS@Ce6@Matrix) cross-linked by chitosan (CS) and oxidized sodium alginate (OSA), co-loaded with Berberine–Asiaticoside cocrystal (BBR-AS) and chlorin e6-loaded chitosan nanoparticles (Ce6@CS NPs). The BBR-AS co-crystal was prepared by solvent method and verified to significantly improve the solubility and dissolution of asiaticoside. The Ce6@CS NPs were fabricated via non-solvent-assisted counterion complexation, showing high encapsulation efficiency, uniform particle size, and efficient singlet oxygen generation under irradiation. The hydrogel exhibited a three-dimensional porous network, favorable rheology, high water content, pH-dependent swelling and erosion behaviors, and significantly promoted BBR/AS release in vitro. In vitro experiments demonstrated strong antibacterial activity against *Escherichia coli* and Staphylococcus aureus, good cytocompatibility, and enhanced migration of L929 and Hacat cells. In a rat infectious wound model, the hydrogel combined with light irradiation markedly accelerated wound closure, promoted collagen deposition and angiogenesis, upregulated VEGF/CD31, and downregulated TNF-α/IL-6. In conclusion, BBR-AS@Ce6@Matrix integrates co-crystal solubilization, nanoparticle-facilitated release, and photodynamic synergy to achieve antibacterial, anti-inflammatory, pro-angiogenic and tissue remodeling effects, providing a promising multifunctional platform for infectious wound repair.

## 1. Introduction

Chronic non-healing wounds refer to areas of tissue damage where, after four weeks of standard treatment, normal repair fails to occur, leaving the healing process arrested in a pathological inflammatory state. Such wounds are commonly seen in patients with chronic diseases or complex injuries, including diabetes mellitus, lower limb varicose veins, paraplegia with prolonged bed rest, etc. [1]. Their pathogenesis is associated with dysregulated inflammation and impaired angiogenesis, and they are characterized by difficult treatment, prolonged disease course, and high medical costs [2]. They not only impair patients’ limb function and quality of life but also predispose to complications such as infection. Moreover, with population aging and shifts in disease patterns, the number of affected patients is increasing annually, making this an important public health issue that urgently needs to be addressed [3].

The key to treating chronic wounds lies in debridement and infection control. However, excessive debridement may aggravate tissue defects, while systemic administration of antibiotics suffers from insufficient local concentration, propensity for drug resistance, and adverse effects on the liver and kidneys [4]. Currently used clinical approaches such as routine dressing changes, intravenous antibiotics, and negative-pressure drainage often fail to stably control infection and yield suboptimal outcomes. In addition, the global overuse of antibiotics has exacerbated the emergence of drug-resistant bacteria, further increasing the difficulty of treating wound infections [5].

The treatment of infected wounds faces multiple challenges, including pathogenic microbial infection, uncontrolled inflammation, and delayed repair. Traditional therapies have drawbacks such as a narrow antibacterial spectrum, drug resistance, and poor healing effects [6]. Therefore, there is an urgent medical need to develop novel therapeutic modalities that combine high antibacterial efficacy, good biocompatibility, and pro-healing functions, and to explore new theories and methods for infected wound repair. Innovative strategies such as harnessing bioactive components from traditional Chinese medicine and developing photodynamic therapy represent cutting-edge research directions.

Berberine (BBR), a major active component of *Coptis chinensis* and *Phellodendron amurense*, exhibits strong antibacterial activity, high biocompatibility, and low propensity to induce drug resistance. It also modulates inflammation and metabolism, and its activity can be further enhanced by structural modification [7,8,9]. Asiaticoside (AS) possesses significant anti-inflammatory, anti-scarring, and wound-healing properties, and can improve the wound repair microenvironment by regulating relevant signaling pathways [10,11,12]. However, the two monomers have inherent defects when applied alone for wound treatment. Single BBR exerts prominent antibacterial effects but lacks targeted wound repair and anti-scarring capacities. In contrast, AS shows excellent efficacy in promoting wound regeneration and inhibiting scar formation, while its clinical application is severely hampered by poor water solubility and negligible antibacterial activity. Considering their complementary pharmacological effects and different solubility characteristics, we innovatively constructed a BBR-AS cocrystal system. The excellent water solubility of BBR acts as a perfect coformer to greatly improve the solubility limitation of poorly soluble AS. Furthermore, the cocrystal integrates the unique advantages of two monomers, realizing synergistic therapeutic effects: it combines the antibacterial property of BBR and the wound repair as well as the anti-scarring functions of AS. Compared with pure BBR or pure AS alone, this dual-drug cocrystal can simultaneously inhibit wound bacterial infection, alleviate inflammatory response and accelerate wound tissue repair, achieving superior comprehensive wound healing outcomes.

Although conventional wound dressings primarily serve to provide a physical barrier against external contaminants, absorb exudate, and maintain a moist environment, they are typically replaced after a limited period and lack active therapeutic functions against established infections or pathological inflammation. In particular, bacterial colonization can rapidly progress to biofilm formation, which not only resists conventional antibiotic treatment but also exacerbates inflammation and delays tissue remodeling. To address these challenges, the incorporation of phototherapy into the wound dressing system offers a distinct advantage. Light-activated therapy, specifically photodynamic therapy (PDT), generates ROS upon irradiation, which can effectively eradicate a broad spectrum of bacteria, including drug-resistant strains, and disrupt biofilm structures. Moreover, PDT has been shown to modulate excessive inflammatory responses and promote angiogenesis, thereby accelerating the healing process. Moreover, as a non-invasive antimicrobial method, PDT offers advantages such as high efficiency and low drug resistance [13,14]. Among photosensitizers, chlorin e6 (Ce6) has excellent photosensitizing properties but poor water solubility. Nano-drug delivery systems can achieve sustained drug release and targeted delivery, increasing local drug concentration. Chitosan (CS), a natural polymer with good biocompatibility and biodegradability, forms nanoparticles that possess both antibacterial activity and drug-delivery potential, making it suitable for constructing local drug delivery systems [15].

The core principle of modern wound repair is to maintain a moist wound environment. Hydrogels, owing to their high water content, good water retention, and ability to form a physical barrier, are ideal wound dressings and drug carriers, enabling local sustained release of active substances and reducing systemic adverse effects [16]. Chitosan and oxidized sodium alginate (OSA) can form a stable composite hydrogel matrix via a Schiff base reaction, providing a reliable scaffold for drug delivery [17]. Combining hydrogels with topical application of traditional Chinese medicine can fully leverage the advantages of Chinese medicine while improving formulation stability and ease of use.

Based on the above background, this project proposes a three-in-one synergistic therapeutic strategy of “Chinese medicine cocrystal–functionalized nanocarrier–photo-responsive hydrogel”. The specific plan is as follows: (1) prepare a BBR-AS cocrystal and characterize it by multiple methods to enhance its solubility and bioavailability; (2) construct Ce6-loaded chitosan nanoparticles (Ce6@CS NPs) to improve the water solubility of Ce6, and characterize their physicochemical properties and photodynamic performance; (3) integrate the above components into a chitosan–OSA crosslinked hydrogel to build an intelligent photo-responsive composite hydrogel (BBR-AS@Ce6@Matrix), systematically characterize its physicochemical properties, and investigate its in vitro drug release, antibacterial efficacy, biosafety, and pro-healing effects; and finally, (4) evaluate its therapeutic effect in vivo using a rat infected wound model, comparing it with clinically used gels, aiming to provide a safe, efficient, and convenient new treatment strategy for refractory infected wounds in clinical practice.

## 2. Results and Discussion

### 2.1. Characterization Results of BBR-AS Cocrystal

**Figure 1:**
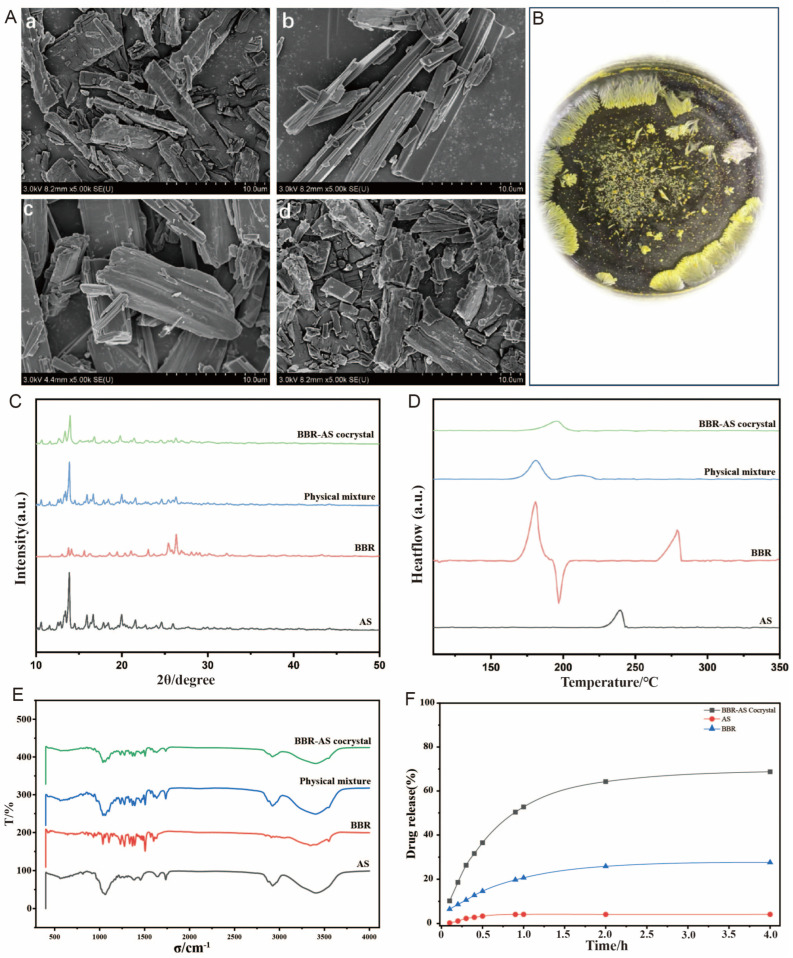
Characterization results of BBR-AS cocrystal. (**A**). SEM image of BBR-AS cocrystal. (**a**) BBR. (**b**) AS. (**c**) Physical mixture of BBR and AS. (**d**) BBR-AS cocrystal. (**B**) Photograph of the BBR-AS cocrystal. (**C**) XRD pattern of the BBR-AS cocrystal. (**D**) DSC pattern of the BBR-AS cocrystal. (**E**) FTIR pattern of the BBR-AS cocrystal. (**F**) Drug Release in BBR-AS cocrystal.

Scanning electron microscopy (SEM) characterization results further confirm that pure BBR (Figure 1A(a)) and AS monomer (Figure 1A(b)) both exhibit regular, dense crystal morphologies, while their physical mixture (Figure 1A(c)) retains the respective crystal characteristics. In contrast, the morphology of the cocrystal system (Figure 1A(d)) undergoes significant changes, with a substantial increase in the proportion of amorphous particles and a marked decrease in crystal integrity and regularity. This phenomenon fully indicates that the molecular-level interactions between the two not only disrupt the lattice arrangement of the original crystals but also induce the formation of a new amorphous phase. Furthermore, the physical image of the cocrystal in Figure 1B intuitively presents the macroscopic morphology of the cocrystal product, whose color and texture differ from those of the pure compounds and the physical mixture. Together with the aforementioned microstructural and morphological characteristics, these observations further support the uniqueness of the BBR-AS cocrystal system in terms of molecular assembly and physical properties.

The melting point determination results (Supplementary Table S1) show that the melting range of BBR is 201.7–203.1 °C, that of AS is 232.2–235.8 °C, while that of BBR-AS is 227.8–229.4 °C. In comparison, the melting range of BBR-AS is significantly different from those of the two parent drugs. The change in melting point is one of the important characteristics of cocrystal formation, as the crystal structure of a cocrystal differs from that of the parent drugs, typically exhibiting new thermodynamic properties. Therefore, combined with the melting point data and crystal morphological characteristics, it can be preliminarily inferred that BBR and AS have successfully formed a cocrystal.

The XRD analysis results (Figure 1C) show that the characteristic peaks of BBR at 14.0° and AS at 20.4°, among others, completely disappear in the diffraction pattern of the BBR-AS cocrystal, while new characteristic peaks appear at distinct diffraction angles. There are significant differences between the diffraction pattern of BBR-AS and those of BBR and AS. These results indicate that BBR and AS have formed a novel crystalline phase structure through cocrystallization, demonstrating the successful synthesis of the BBR-AS cocrystal. The SEM images show an increased population of irregular particulate domains with indistinct edges, which are morphologically consistent with amorphous-like particles. This morphological observation, when correlated with the concurrent suppression of crystalline Bragg peaks and the emergence of a broad diffuse scattering halo in the corresponding XRD patterns, collectively indicates a substantial increase in the proportion of amorphous material in the cocrystal system.

The DSC analysis results (Figure 1D) further demonstrate that the BBR-AS cocrystal exhibits a distinct endothermic peak at 195.8 °C, which lies between those of BBR (180.8 °C) and AS (238.7 °C) and is different from the thermal behaviors of both. This indicates that BBR-AS is neither a single component nor a simple physical mixture, but rather a new substance with unique thermodynamic properties.

Finally, the molecular vibrational characteristics of the cocrystal were characterized by FTIR. The results (Figure 1E) show that BBR and AS exhibit multiple characteristic absorption peaks in their infrared spectra, reflecting the presence and vibrational modes of functional groups such as hydroxyl, aromatic ring, carbonyl, and ether bonds in their molecules. The main absorption peaks of BBR are concentrated around 3400 cm^−1^ (O-H), 2920 cm^−1^ and 2850 cm^−1^ (C-H), 1600 cm^−1^ and 1500 cm^−1^ (C=C), and 1250 cm^−1^ and 1050 cm^−1^ (C-O/C-N); the main absorption peaks of AS are concentrated around 3400 cm^−1^ (O-H), 2920 cm^−1^ and 2850 cm^−1^ (C-H), 1700 cm^−1^ (C=O), 1600 cm^−1^ and 1500 cm^−1^ (C=C), and 1250 cm^−1^ and 1050 cm^−1^ (C-O/C-N). As shown in Figure 1E, the FTIR spectrum of the BBR-AS cocrystal exhibits new characteristic absorption peaks. Some characteristic peaks of the individual drugs, such as the O-H stretching vibration peak (around 3400 cm^−1^), show shifts and intensity changes, indicating that BBR and AS have established new intermolecular interactions through hydrogen bonding. The shifts and intensity changes in the C=O and C=C stretching vibration peaks reflect the presence of π-π stacking or other non-covalent interactions between the aromatic rings and carbonyl groups. These changes indicate that BBR and AS have formed a new chemical structure in the cocrystal, rather than a simple physical mixture. No obvious impurity peaks are observed in the FTIR spectrum, indicating high purity and chemical stability of the cocrystal. The infrared characterization results reveal the structural characteristics of the BBR-AS cocrystal at the molecular vibrational level, demonstrating the successful formation of the cocrystal.

On the basis of the successful preparation of the BBR-AS cocrystal, the solubility of the poorly soluble AS in the cocrystal was determined. The results, as shown in Supplementary Table S2, indicate that the solubility of AS pure drug is only 61.18 μg/mL, whereas the solubility of AS in the cocrystal is increased to 416.90 μg/mL, which is approximately 6.8 times that of the parent drug. This demonstrates that cocrystallization can significantly enhance the solubility of AS. Further determination of the in vitro release behavior of AS, BBR, and their cocrystal (Figure 1F) revealed that the cumulative release rate of the AS component in BBR-AS is significantly higher than that of AS pure drug. At 4 h, the cumulative dissolution rate of AS in BBR-AS is nearly 80%, while that of AS pure drug is approximately 5%, indicating that cocrystallization significantly improves the release of AS.

### 2.2. Characterization Results of Ce6@CS NPs

**Figure 2:**
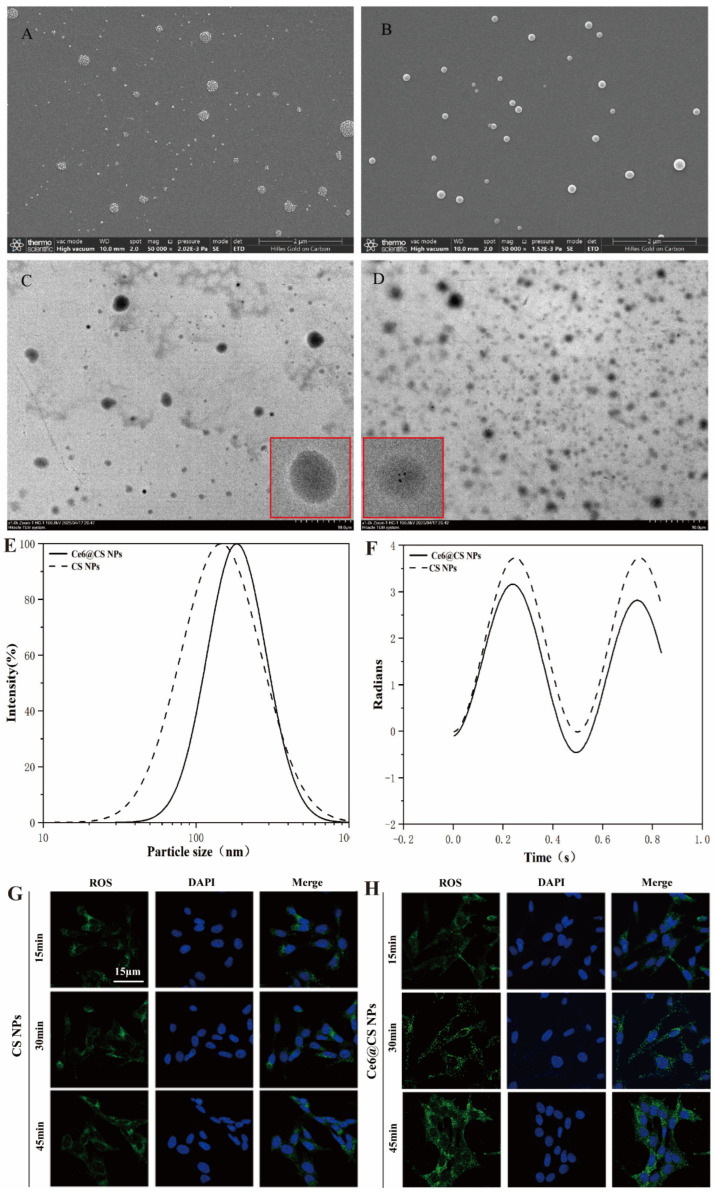
Characterization results of Ce6@CS NPs. (**A**) SEM image of blank CS NPs. (**B**) SEM image of Ce6@CS NPs. (**C**) TEM image of blank CS NPs. (**D**) TEM image of Ce6@CS NPs. (**E**) Particle size distribution and (**F**) zeta potential distribution of Ce6@CS NPs and CS NPs. (**G**,**H**) Detection of the intracellular ROS generation-promoting ability of Ce6@CS NPs in L929 cells.

The SEM and TEM characterization results of Ce6@CS NPs are shown in Figure 2A–D. Blank CS NPs exhibit a regular spherical or sphere-like structure. After loading Ce6 to form the Ce6@CS NPs composite system, the morphology shows no significant change and retains the original spherical or sphere-like characteristics. This result indicates that chitosan nanoparticles can serve as a stable carrier to effectively encapsulate Ce6, and the loading process does not significantly affect their structural morphology, further confirming their feasibility and structural stability as a drug delivery vehicle. The average particle size of CS NPs is 147.47 ± 7.38 nm, with a zeta potential of 31.25 ± 1.48 mV, while that of Ce6@CS is 184.1 ± 3.38 nm, with a zeta potential of 29.14 ± 5.42 mV (Supplemental Table S3). As shown in Figure 2E,F, compared with CS NPs, the particle size of Ce6@CS is significantly increased, indicating that the nanoparticles formed by crosslinking chitosan and sodium tripolyphosphate have successfully encapsulated Ce6. The singlet oxygen generation capacity of Ce6@CS was examined. As shown in Supplemental Figure S1, under 660 nm laser irradiation, free Ce6 rapidly generates singlet oxygen within 1 to 10 min. In contrast, Ce6@CS slowly generates singlet oxygen within 1 to 10 min, with higher efficiency than free Ce6. This indicates that after being encapsulated by chitosan, Ce6 exhibits better photodynamic efficacy.

To evaluate the ability of Ce6@CS NPs to generate ROS in living cells, CS NPs and Ce6@CS NPs were incubated with L929 cells, followed by irradiation with a 660 nm laser for 15, 30, and 45 min, respectively. The results (Figure 2G,H) showed that the intracellular ROS fluorescence intensity in the Ce6@CS NPs-treated group was significantly higher than that in the CS NPs-treated group, indicating that laser-excited Ce6 can effectively generate ROS in living cells. This result is consistent with the singlet oxygen detection findings, further validating the photodynamic effect of Ce6@CS NPs.

### 2.3. Characterization Results of the Hydrogel

**Figure 3:**
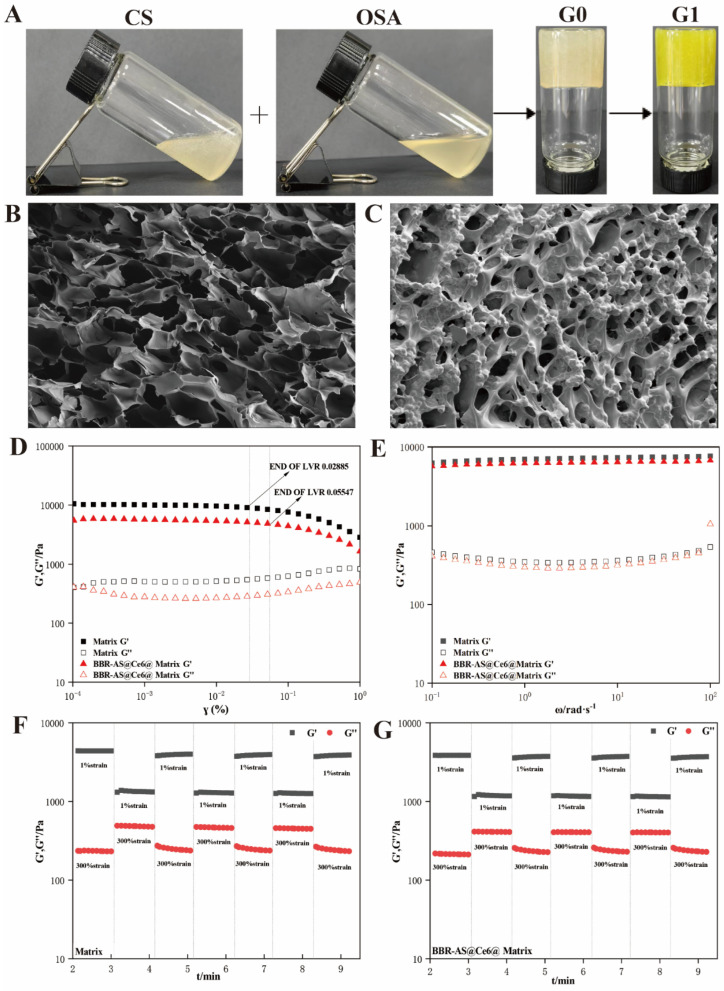
Characterization results of BBR − AS@Ce6@Matrix hydrogel. (**A**) Hydrogel formation diagram. (**B**) SEM images of hydrogel. (**C**) SEM images of hydrogel loaded with BBR-AS cocrystal and Ce6 NPs. (**D**) Amplitude sweep. (**E**) Frequency sweep. (**F**) Strain recovery sweep of Matrix. (**G**) Strain recovery sweep of BBR − AS@Ce6@Matrix hydrogel.

The hydrogel in this study was formed by crosslinking between CS and OSA, both of which are naturally biodegradable polymers with good biocompatibility. The crosslinking reaction between CS and OSA was found to be pH-dependent. Under neutral to acidic conditions, a Schiff base reaction occurred between the amino groups on CS and the aldehyde groups on OSA, resulting in the formation of imine bonds and a crosslinked structure. Concurrently, intermolecular hydrogen bonding contributed to the formation of a crosslinked network, promoting hydrogel formation, as illustrated in Figure 3A.

As shown by the SEM analysis, the prepared hydrogel (Matrix) (Figure 3B) exhibited a three-dimensional porous network structure with a uniform pore size distribution (approximately 40 μm), consisting of irregular polygonal or elliptical pores. The pore walls were smooth and highly interconnected, forming a continuous and permeable scaffold morphology with excellent internal connectivity and mechanical stability. The hydrogel was considered to provide ample specific surface area and space for drug loading, facilitating the diffusion and transport of liquids and drug molecules. The drug-loaded hydrogel (BBR-AS@Ce6@Matrix) displayed a structure consistent with that of the blank hydrogel (Matrix), and Ce6@CS NPs were clearly observed to be distributed within the hydrogel (Figure 3C), confirming that Ce6@CS NPs were successfully encapsulated in the porous network structure.

Rheological property analysis revealed that the hydrogel exhibited a favorable amplitude sweep curve (Figure 3D), maintaining structural integrity under a wide range of applied strains without disruption, demonstrating strong resistance to external force interference. This suggested its structural stability in dynamic or wide-range external force scenarios, such as drug delivery systems and flexible biomedical devices. The angular frequency sweep curve (Figure 3E) showed that Matrix maintained a high storage modulus (G′) of approximately 10^4^ Pa over a broad angular frequency range, with almost no significant attenuation as the frequency changed, indicating that its elastic network structure was very stable, and the frequency had minimal impact on its elastic contribution. The G′ of BBR-AS@Ce6@Matrix was also on the order of 10^4^ Pa, with no significant difference from that of Matrix across the entire frequency range, indicating that the elastic network strength of the hydrogel was not significantly weakened after loading BBR-AS and Ce6@CS. The loss modulus (G″) (viscous behavior) of Matrix was found to be much lower than its G′, remaining below the order of 10^3^ Pa, indicating that the material was predominantly elastic. Moreover, G″ showed almost no significant fluctuation with frequency changes, indicating stable viscous contribution. BBR-AS@Ce6@Matrix also exhibited predominantly elastic behavior.

The strain cycle results presented in Figure 3F,G indicated that both hydrogels maintained G′ > G″ at all stages, demonstrating that even after disruption by a large strain (300%), their “elastic-dominated gel nature” remained unchanged, and the network structure remained centered on elastic crosslinking. Both hydrogels exhibited recoverable elastic network properties during strain cycling, suggesting that they possessed self-healing capability after deformation, making them suitable for use in drug delivery systems.

### 2.4. Characterization Results of the Hydrogel’s Water Retention, Swelling, Erosion, and Drug Release Properties

**Figure 4:**
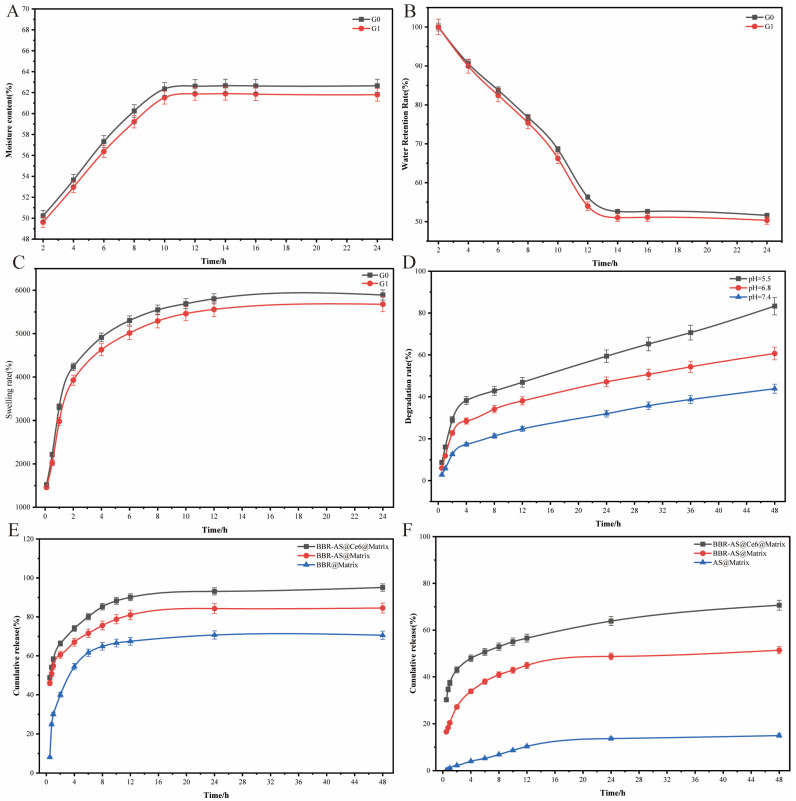
Characterization results of the hydrogel’s water retention, swelling, erosion, and drug release properties. (**A**) Test results for the moisture content of hydrogels. (**B**) Test results for the water retention rate of hydrogels. (**C**) Test results for the swelling rate of hydrogels. (**D**) Test results for the degradation of hydrogels. (**E**) The cumulative release of BBR within 48 h. (**F**) The cumulative release of AS within 48 h. Data are presented as mean ± SD (*n* = 3 independent replicates per group at each time point).

Both G0 and G1 hydrogels prepared in this study exhibited typical water-absorbing swelling behavior. The water content of both groups was found to increase rapidly over time and then stabilize at approximately 62–63% after 12 h (Figure 4A). Meanwhile, the water retention rate decreased rapidly during the first 12 h and subsequently stabilized, ultimately maintaining at about 50% (Figure 4B). The equilibrium water content and swelling ratio of G0 were slightly higher than those of G1, indicating stronger hydrophilic characteristics. The swelling ratio curves also showed a trend of rapid initial increase followed by a plateau reached at 12 h, with a maximum swelling ratio of approximately 600% for G0, which was slightly higher than that of the G1 group (approximately 560%), as shown in Figure 4C.

Degradation experiments demonstrated that the hydrogels exhibited significant pH-responsive behavior (Figure 4D). The fastest degradation rate was observed at pH 5.4 (simulating the acidic microenvironment of wounds), where the degradation rate reached nearly 80% at 48 h, which was significantly higher than those observed at pH 6.8 (approximately 60%) and pH 7.4 (approximately 40%), meeting the microenvironmental requirements of inflamed wounds.

The results of drug release experiments showed that all three groups of drug-loaded hydrogels (BBR-AS@Ce6@Matrix, BBR-AS@Matrix, and AS@Matrix) exhibited a characteristic of rapid initial release followed by slow sustained release. Among them, the cumulative release rate of BBR-AS@Ce6@Matrix was consistently the highest, reaching approximately 95% and 75% at 48 h in the two release curves, respectively, which were significantly higher than those of BBR-AS@Matrix (approximately 80% and 55%) and AS@Matrix (approximately 70% and 15%), as shown in Figure 4E,F. These results indicated that the introduction of Ce6 and the BBR-AS cocrystal form synergistically enhanced the drug release efficiency, achieving both rapid initial release and sustained long-term release, thereby providing efficient and long-lasting drug delivery support for wound healing.

### 2.5. Intracellular ROS Generation in L929 Cells Was Promoted by the Ce6@CS NPs-Loaded Hydrogel

**Figure 5:**
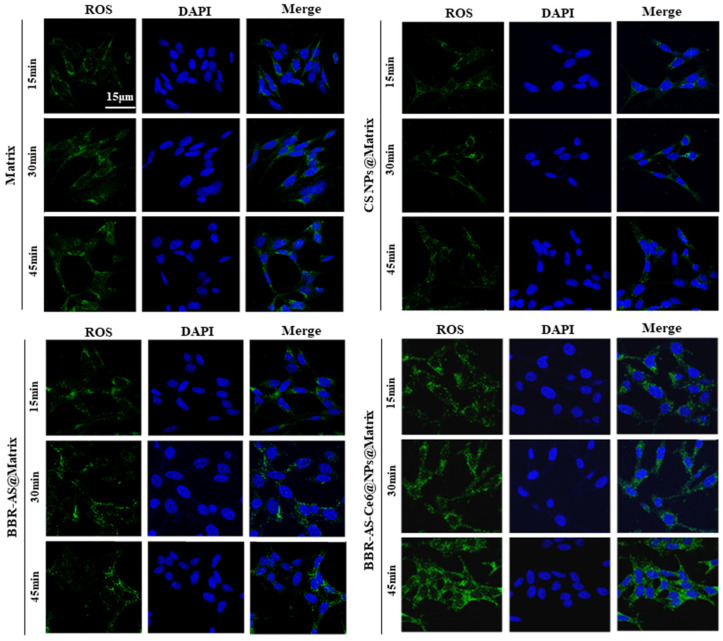
BBR-AS@Ce6@Matrix promotes ROS production in L929 cells.

To verify that the Ce6-loaded hydrogel could also generate ROS in living cells, differences in ROS production among the blank matrix hydrogel (Matrix), the CS NPs-loaded matrix hydrogel (CS NPs@Matrix), the BBR-AS cocrystal-loaded hydrogel (BBR-AS@Matrix), and the BBR-AS and Ce6@CS NPs-loaded composite hydrogel (BBR-AS@Ce6@Matrix) were compared in L929 cells. The results are shown in Figure 5. Significantly higher ROS fluorescence intensities were observed in L929 cells treated with BBR-AS@Ce6@Matrix at all time points compared to the other hydrogel groups. Moreover, ROS production was found to increase in a time-dependent manner with prolonged 660 nm laser irradiation. These findings demonstrated that Ce6, once loaded into the hydrogel, could still effectively generate ROS in living cells, thereby validating the photodynamic effect of BBR-AS@Ce6@Matrix.

### 2.6. Inhibitory Effect of the Drug-Loaded Hydrogel Against Escherichia coli and Staphylococcus aureus

**Figure 6:**
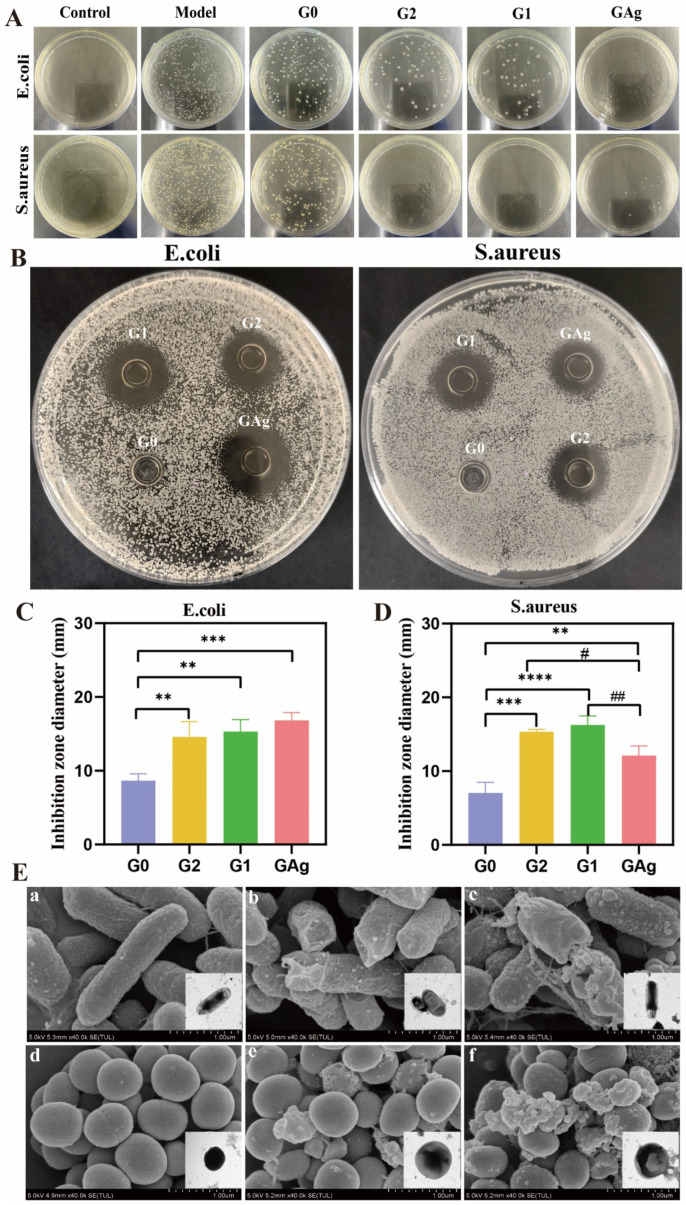
The inhibitory effect of the hydrogel on *Escherichia coli* and Staphylococcus aureus. (**A**) The antibacterial activity of the hydrogel was evaluated using the plate counting method. (**B**) The antibacterial activity of the hydrogel was evaluated using the oxford cup method. (**C**) The comparison of the inhibition zone diameters of *Escherichia coli* among different experimental groups. (**D**) The comparison of the inhibition zone diameters of *Staphylococcus aureus* among different experimental groups. (**E**) SEM and TEM images of *Escherichia coli* and *Staphylococcus aureus* before and after treatment with different hydrogels. (**a**,**d**). Blank control group. (**b**,**e**) BBR-AS@Matrix treatment group. (**c**,**f**) BBR-AS@Ce6@Matrix+ laser excitation group. Note: Control group: Blank culture medium (no treatment). Model group: Model group (no intervention). G0 group: Matrix hydrogel group. G1 group: BBR-AS@Ce6@Matrix hydrogel group. G2 group: BBR-AS@Matrix hydrogel group. GAg group: Positive control group. The results represent the mean ± SD of 3 independent experiments. ** *p* < 0.01, *** *p* < 0.001, **** *p* < 0.0001 vs. G0; ^#^ *p* < 0.05, ^##^ *p* < 0.01 vs. GAg.

Wound exudate provides a moist environment that facilitates bacterial proliferation, and controlling local infection was considered a crucial step in preventing further disease progression. In this study, the antibacterial activity of the hydrogels was first evaluated using the plate counting method. The results (Figure 6A) showed that when the bacterial inoculation density was 10^5^ CFU/mL, broad-spectrum antibacterial activity was exhibited by G0, G1, G2, and GAg compared with the Model group. (1) For *E. coli*: Compared with the Model group, *E. coli* colony formation was inhibited by G0, indicating certain antibacterial activity. Compared with G0, bacterial colony formation was significantly reduced by both G1 and G2. Relative to the positive control group GAg, the antibacterial performance of G1 and G2 against *E. coli* was slightly weaker. (2) For *S. aureus*: Compared with the Model group, *S. aureus* colony formation was inhibited by G0, indicating certain antibacterial activity. Compared with G0, bacterial colony formation was significantly reduced by both G1 and G2. Relative to the positive control group GAg, better antibacterial performance against *S. aureus* was exhibited by G1 and G2.

The antibacterial performance of the different hydrogel groups against *E. coli* and *S. aureus* was further investigated using the Oxford cup method. The results (Figure 6B) showed that excellent antibacterial performance against both *E. coli* and *S. aureus* was exhibited by the G1, G2, and GAg groups (*p* < 0.01), with obvious inhibition zones being formed. In contrast, almost no inhibition zone was formed by the G0 group, indicating that the Matrix hydrogel itself possessed weak antibacterial activity. This further confirmed that the antibacterial effect was primarily derived from the loaded BBR-AS cocrystal and Ce6@CS nanoparticles in the hydrogels. Differences in antibacterial activity were observed among the different groups, as shown in Supplementary Table S4, Figure 6C,D, which was manifested as significant differences in inhibition zone diameters: (1) Against *E. coli*, the order of inhibition zone diameters was GAg > G1 > G2 > G0. The best antibacterial activity was exhibited by the GAg group, with the largest inhibition zone diameter being formed. Slightly weaker antibacterial activity was exhibited by the G1 group, which was marginally lower than that of the GAg group but significantly higher than that of the G2 group, indicating that the antibacterial capacity against *E. coli* could be enhanced by the introduction of Ce6@CS nanoparticles into the hydrogel. (2) Against *S. aureus*, the order of inhibition zone diameters was G1 > G2 > GAg > G0, which was significantly different from that observed for *E. coli*. The best antibacterial activity was exhibited by the G1 group, whose inhibition zone diameter was significantly larger than those of the G2 and GAg groups, while weaker antibacterial activity was exhibited by GAg compared with the G1 and G2 groups (*p* < 0.05).

In the G1 group, which was loaded with both BBR-AS cocrystal and Ce6@CS nanoparticles, a synergistic antibacterial effect was exerted by the two components. Moreover, drug release was promoted by the Ce6@CS nanoparticles, thereby enhancing the diffusion efficiency of the antibacterial components. In the G2 group, which was loaded only with the BBR-AS cocrystal, lower drug release efficiency was observed compared with the G1 group, resulting in slightly weaker antibacterial activity. Silver ion hydrogel, which is a commonly used clinical antibacterial material, exhibited more prominent antibacterial efficacy against the Gram-negative bacterium *E. coli*, whereas its antibacterial activity against the Gram-positive bacterium *S. aureus* was inferior to that of the G1 and G2 groups. These findings demonstrated that the G1 composite hydrogel constructed in this study possessed broader and more balanced antibacterial potential against clinically common pathogenic bacteria, providing important in vitro experimental support for its application in the treatment of infected wounds.

The microstructures of the two bacterial species were examined by SEM and TEM. As shown in Figure 6E(a), the untreated control group of *E. coli* exhibited intact morphological structures, presenting a typical rod shape with slight surface wrinkles. Meanwhile, *S. aureus* in the control group (Figure 6E(d)) displayed a uniform, smooth spherical structure with clear cell contours. After being treated with the BBR-AS@Matrix hydrogel, the morphology of both bacterial species was found to be obviously deformed, although their basic contours remained identifiable, as shown in Figure 6E(b,e). Subsequently, when both bacterial species were treated with the BBR-AS@Ce6@Matrix hydrogel combined with light irradiation, their cellular structures were significantly disrupted, and leakage of bacterial contents was observed, as shown in Figure 6E(c,f). These results indicated that stronger destructive effects against both bacterial species were exerted by the combined action of the BBR-AS cocrystal and Ce6@CS nanoparticles.

Due to the absence of a control group consisting of the matrix loaded exclusively with Ce6 under irradiation, the observed enhancement in antibacterial activity cannot be conclusively attributed to a true synergistic interaction between Ce6 and the BBR-AS cocrystal, as the contribution of Ce6-mediated photodynamic therapy alone cannot be fully excluded. Future studies will include appropriate controls to rigorously validate the synergistic effect.

### 2.7. Cell Migration-Promoting Effect of the Hydrogel

**Figure 7:**
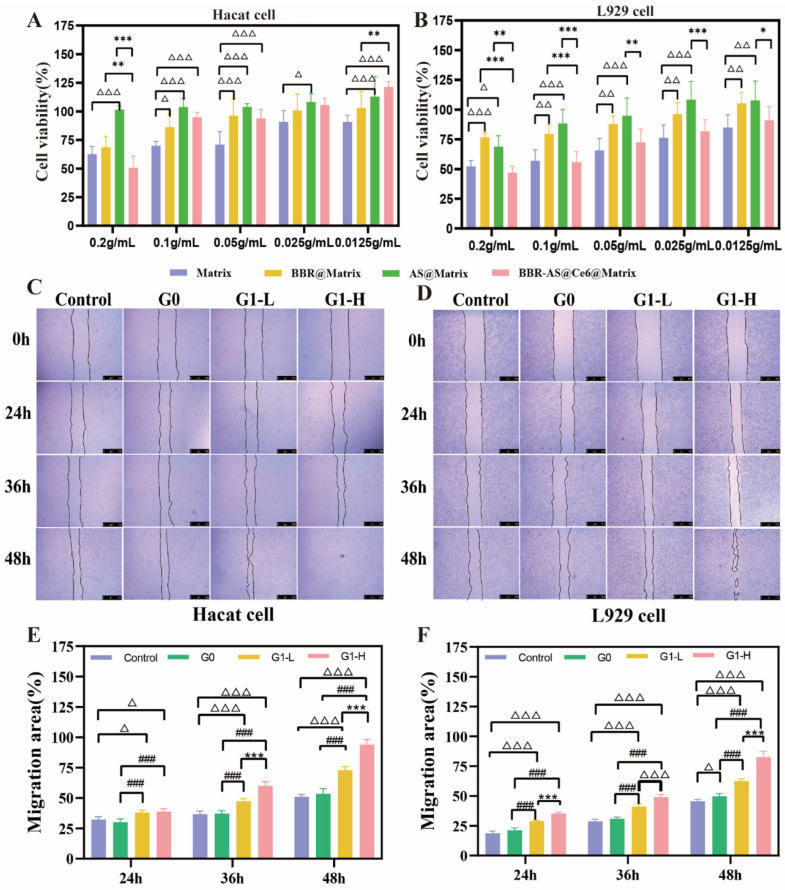
The hydrogel promotes the migration of HaCaT cells and L929 cells. (**A**) Safety evaluation of the hydrogel on HaCaT cells. (**B**) Safety evaluation of the hydrogel on L929 cells. (**C**) Hydrogel promotes the migration of HaCaT cells. (**D**) Hydrogel promotes the migration of L929 cells. (**E**) Statistical graph of HaCaT cell migration. (**F**) Statistical graph of l929 cell migration. The results represent the mean ± SD of 3 independent experiments. ^△^ *p* < 0.05, ^△△^ *p* < 0.01, ^△△△^ *p* < 0.001, vs. Control group, ^###^ *p* < 0.001, vs. G0, * *p* < 0.05, ** *p* < 0.01, *** *p* < 0.001, vs. G1-L group.

The cytotoxicity of the blank hydrogel (Matrix), BBR@Matrix (G3), AS@Matrix (G4), and BBR-AS@Ce6@Matrix (G1) was evaluated in HaCaT and L929 cells using the CCK-8 assay. The results (Figure 7A,B) showed that within the concentration range of 0.0125–0.2 g/mL, the cell viabilities of both cell lines treated with the hydrogel extracts of all groups were above 50%. Compared with the single-drug-loaded hydrogels, slightly higher cytotoxicity was exhibited by the G1 group. At a concentration of 0.025 g/mL, the cell viability of HaCaT cells in the G1 group was 105.410 ± 6.052%, and that of L929 cells was 81.792 ± 9.776%, indicating that proliferative promotion effects on both cell lines were exerted by the hydrogel extract at this concentration. Based on the above results, two concentrations of hydrogel extracts, 0.025 g/mL (G1-H) and 0.0125 g/mL (G1-L), were selected for subsequent scratch wound healing assays to evaluate cell migration ability.

Cell migration is considered an indispensable and critical step in the wound healing process. As shown in Figure 7C–F, the scratch wound healing assay results revealed that during the 24 to 48 h culture period, significantly enhanced migration abilities were observed in both HaCaT and L929 cells treated with the high-concentration (G1-H) and low-concentration (G1-L) extracts of the BBR-AS@Ce6@Matrix hydrogel compared with the control group and the blank hydrogel matrix group (G0) (*p* < 0.001). These findings indicated that no significant promotion of cell migration was exerted by the hydrogel matrix alone, whereas cell migration was significantly accelerated by the G1 group in both cell lines, thereby demonstrating that this drug-loaded system possesses favorable application potential for promoting wound healing.

### 2.8. Promotion of Infected Wound Healing in Rats by the Hydrogel

**Figure 8:**
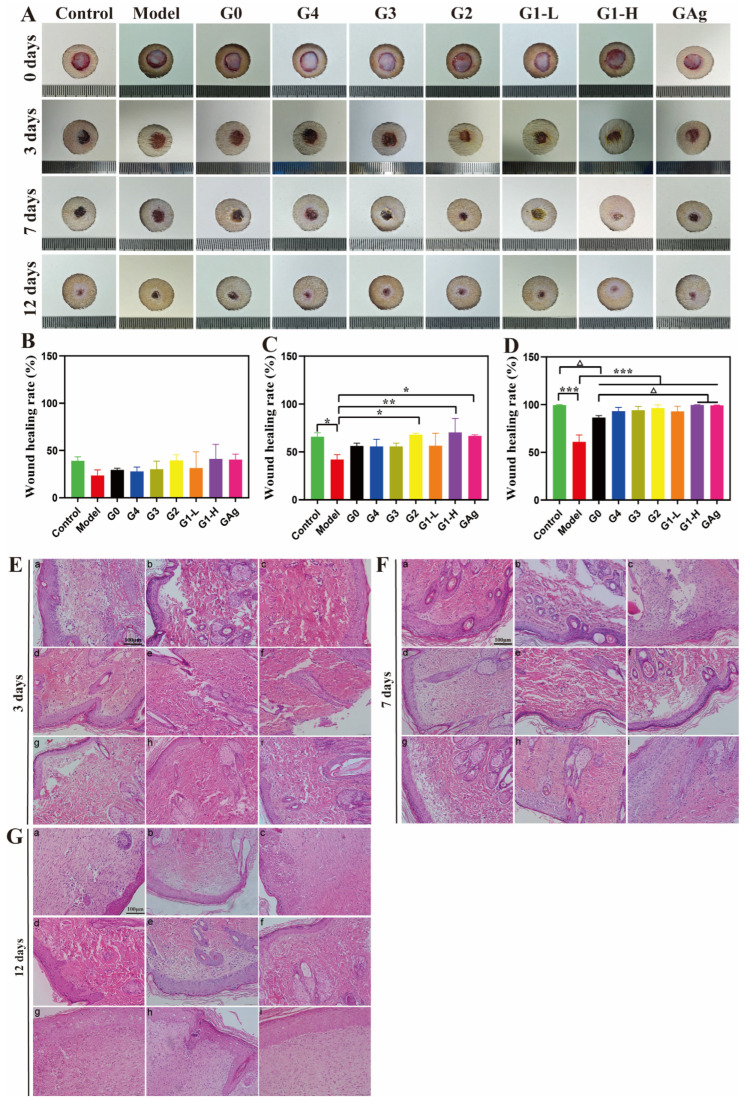
The hydrogel promotes the healing of infected wounds in rats. (**A**) Healing trajectory of infected wounds in rats on days 3, 7, and 12. (**B**) Wound healing rate on day 3 in rats. (**C**) Wound healing rate on day 7 in rats. (**D**) Wound healing rate on day 12 in rats. (**E**–**G**). Comparison of skin tissue histomorphology among different experimental groups on days 3, 7, and 12 in rats. (**a**) Control group. (**b**) Model group. (**c**) GAg group. (**d**) G0 (Matrix group). (**e**) G3 (BBR@Matrix group). (**f**) G4 (AS@Matrix group). (**g**) G2 (BBR-AS@Matrix group). (**h**) G1-L (BBR-AS@Ce6@Matrix low concentration group). (**i**) G1-H (BBR-AS@Ce6@Matrix high concentration group). The results represent the mean ± SD of 6 independent experiments. * *p* < 0.05, ** *p* < 0.01, *** *p* < 0.001, vs. Model group, ^△^ *p* < 0.05 vs. G0 group.

Wound healing trajectories (Figure 8A) and healing rate statistics (Figure 8B–D) were analyzed across different time points. On day 3, no significant differences in healing rates were observed among all groups. On day 7, the G2, G1-H, GAg, and Control groups exhibited significantly higher healing rates than the other groups, with the G1-H group reaching 70.26 ± 14.56% compared with 42.10 ± 5.11% in the Model group (Figure 8B). By day 12, all treatment groups showed significantly accelerated wound closure relative to the Model group (*p* < 0.001; Figure 8C,D). Importantly, all drug-loaded groups demonstrated superior healing rates compared with the blank matrix (G0) group (*p* < 0.05), confirming that the therapeutic effect was attributable to the active components rather than the matrix alone. Notably, the G1-H group achieved a healing rate of 99.74 ± 0.25% by day 12, which was comparable to that of the GAg group (99.23 ± 0.39%) and significantly higher than that of the G0 group (*p* < 0.05), indicating that the BBR-AS@Ce6@Matrix hydrogel combined with light irradiation achieved nearly complete wound closure with efficacy equivalent to the commercially available silver ion disinfectant gel.

### 2.9. Significant Improvement of Skin Pathological Morphology in Infected Wound Rats by the Hydrogel

Significant time-dependent differences in pathological morphology and therapeutic effects were observed among the experimental groups at different time points (3, 7, and 12 d), with the best therapeutic effects being exhibited by the G1-H and G1-L groups. The detailed findings are as follows:

As shown in Figure 8E–G, on day 3 (early inflammatory phase): In the Control group (sham-operated group), intact skin structure was observed, with clear epidermal and dermal layers and no inflammatory infiltration. In the Model group, marked epidermal erosion, dermal congestion and edema, extensive infiltration of inflammatory cells (neutrophils and macrophages), and severe local tissue damage were observed. In the GAg (positive control), G1-H, G1-L, G2, G3, and G4 groups, varying degrees of inflammation alleviation, reduced epidermal lesion area, and decreased inflammatory cell infiltration were observed. However, significantly greater inflammation relief was exhibited by the G1-H and G1-L groups compared with the other groups, with lower inflammatory infiltration density and a more pronounced epidermal repair trend. No significant inflammation improvement was observed in the G0 group (blank matrix group), with only minor differences being found compared with the Model group.

On day 7 (inflammation resolution and repair phase): In the Model group, relatively severe inflammation was still observed, with no obvious healing of the epidermal defect and considerable residual inflammatory cells in the dermal layer. In the GAg group, further inflammation resolution was observed, with epidermal regeneration being initiated and collagen fiber arrangement in the dermal layer gradually becoming organized. In the G1-H and G1-L groups, inflammation was found to be largely controlled, with the epidermal defect being significantly reduced and abundant newly formed collagen fibers being observed in the dermal layer, where the tissue arrangement tended toward normalization. Slightly better repair efficacy was exhibited by the G1-H group compared with the G1-L group. In the G2, G3, and G4 groups, moderate inflammation resolution was observed, with slower epidermal regeneration and relatively sparse collagen fiber arrangement. In the G0 group, notable residual inflammation was still observed, and tissue repair progressed slowly.

On day 12 (tissue remodeling phase): In the Model group, incomplete skin damage repair was observed, with an still incomplete epidermal structure and a relatively high degree of dermal fibrosis. In the GAg group, the skin structure was found to be essentially restored to normal, with intact epidermal keratinization and tightly and uniformly arranged dermal collagen fibers. In the G1-H and G1-L groups, the skin histological structure was observed to be close to normal, with clear epidermal layering and abundant newly formed dermal collagen fibers. A significantly lower degree of tissue fibrosis was exhibited by these groups compared with the other groups, with the best tissue repair effect being observed in the G1-H group, whose dermal structure was closest to that of the Control group. In the G2, G3, and G4 groups, moderate skin repair was observed, with epidermal regeneration being essentially completed, although the arrangement of collagen fibers remained relatively disorganized. In the G0 group, no obvious skin damage repair was observed, with severe tissue fibrosis being noted and only minor differences being found compared with the Model group.

### 2.10. Promotion of Wound Hair Follicle and Collagen Tissue Regeneration in Rats by the Hydrogel

**Figure 9:**
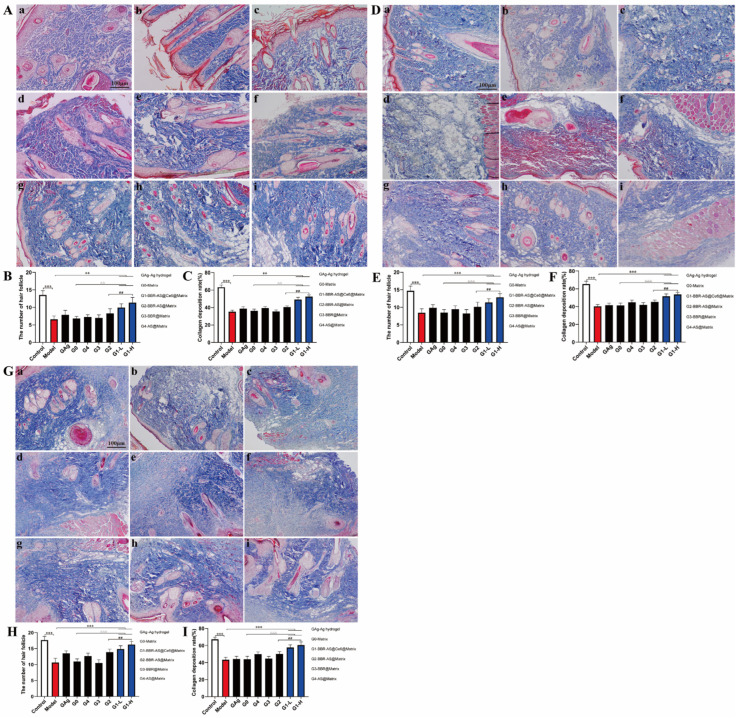
Masson staining results of rat skin tissue after 3, 7 and 12 days of treatment. (**A**) Representative images of Masson staining results after 3 days of treatment. (**B**) Statistical graph of hair follicle count results after 3 days of treatment. (**C**) Statistical graph of collagen deposition rate results after 3 days of treatment. (**D**) Representative images of Masson staining results after 7 days of treatment. (**E**) Statistical graph of hair follicle count results after 7 days of treatment. (**F**) Statistical graph of collagen deposition rate results after 7 days of treatment. (**G**) Representative images of Masson staining results after 12 days of treatment. (**H**) Statistical graph of hair follicle count results after 12 days of treatment. (**I**) Statistical graph of collagen deposition rate results after 12 days of treatment. (**a**) Control group. (**b**) Model group. (**c**) GAg (positive control group). (**d**) G0 (blank matrix group, Matrix). (**e**) G3 (BBR@Matrix group). (**f**) G4 (AS@Matrix group). (**g**) G2 (BBR-AS@Matrix group). (**h**) G1-L (BBR-AS@Ce6@Matrix low-concentration group). (**i**) G1-H (BBR-AS@Ce6@Matrix high-concentration group). The results represent the mean ± SD of 6 independent experiments. ** *p* < 0.01, *** *p* < 0.001 vs. Model group, ^△△^ *p* < 0.01, ^△△△^ *p* < 0.001 vs. Matrix group, ^##^ *p* < 0.01, vs. G2 group.

As shown in Figure 9A–C, on day 3: Intact skin structure was observed in the sham-operated group (Control group), where numerous morphologically normal hair follicles were visible, and densely and regularly arranged deep blue collagen fibers were observed in the dermal layer. Compared with the Control group, a significantly reduced number of hair follicles (*p* < 0.001) and a significantly decreased collagen deposition rate (*p* < 0.001) were observed in the Model group. Extensive inflammatory necrotic areas were noted, with the original collagen structure being disrupted and fragmented, and only a small amount of sparse, lightly stained newly formed collagen fibers was observed. Varying degrees of newly formed collagen were observed in all treatment groups, although hair follicle regeneration had not yet been initiated. Among these groups, the most abundant and relatively orderly arranged newly formed collagen fibers were observed in the G1-L and G1-H groups, with significantly higher collagen deposition rates being found compared with the Model group (*p* < 0.01), the G0 group (*p* < 0.05), and the G2 group (*p* < 0.05). Superior collagen regeneration trends relative to the G0 group were also exhibited by the G2 and G4 groups; however, gaps in collagen fiber density and maturity were still observed compared with the G1-H group (*p* < 0.05). Similar effects were exhibited by the G3 and G0 groups, with only limited improvement being observed.

On day 7 of the experiment, the Model group was still in the inflammation and repair phase, where a small number of immature hair follicle structures were observed to begin forming, and increased but still sparse and disorganized collagen deposition was noted. Differences in hair follicle regeneration and collagen remodeling among the treatment groups began to emerge. Significantly superior outcomes in terms of newly formed hair follicle count (*p* < 0.01) and collagen deposition rate (*p* < 0.01) were exhibited by both the G1-H and G1-L groups compared with the Model group. Faster hair follicle structure recovery and more mature collagen fibers (more tightly arranged with deeper blue staining) were demonstrated by both the G1-H and G1-L groups compared with the G0 and G2 groups (*p* < 0.01). Better outcomes in terms of hair follicle count and collagen deposition were also exhibited by the G2 and G4 groups relative to the G0 group; however, the integrity of hair follicle structures and the maturity of collagen fibers remained inferior to those observed in the G1-H and G1-L groups. Moderate repair effects were shown by the GAg group. The results are presented in Figure 9D–F.

On day 12, incomplete skin repair was observed in the Model group, where a small number of regenerated hair follicles were visible, although their density remained far below that of normal skin. Although increased collagen deposition was noted, it still consisted predominantly of relatively thin type III collagen with disorganized arrangement. The best tissue remodeling effects were exhibited by the G1-H and G1-L groups: hair follicle counts were restored to near-normal levels, with highly statistically significant differences being observed compared with the Model, G0, and G2 groups (*p* < 0.01); the highest collagen deposition rate was achieved, with thick, dense, and orderly arranged blue collagen fibers being observed, presenting a mature morphology predominantly composed of type I collagen, which was significantly superior to all other treatment groups. Significantly superior outcomes in terms of both hair follicle count and collagen maturity were exhibited by the G2 group compared with the G3 and G4 groups (*p* < 0.05), demonstrating the synergistic advantage of the cocrystal. The results are presented in Figure 9G–I.

### 2.11. Effects of the Hydrogel on VEGF, CD31, IL-6, and TNF-α in the Skin of Rats with Infected Wounds

**Figure 10:**
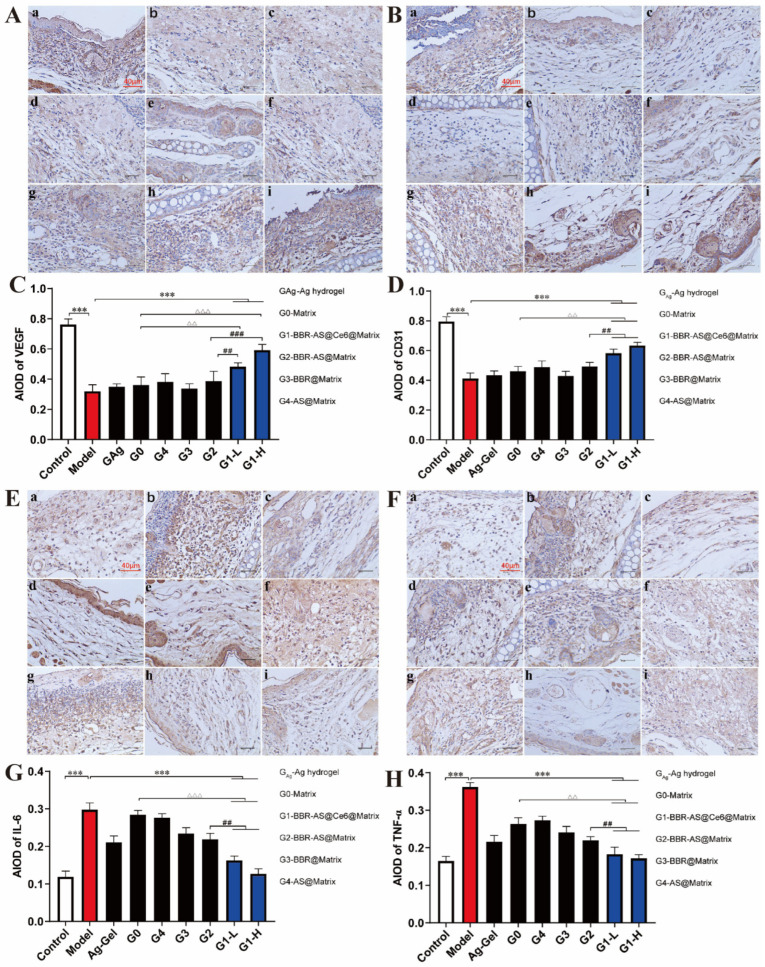
Immunohistochemical detection of VEGF, CD31, IL-6 and TNF-α in rat skin. (**A**) Immunohistochemical detection results of VEGF in rat skin. (**B**) Immunohistochemical detection results of CD31 in rat skin. (**C**) Statistical graph of AIOD for VEGF immunohistochemical detection. (**D**) Statistical graph of AIOD for CD31 immunohistochemical detection. (**E**) Immunohistochemical detection results of IL-6 in rat skin. (**F**) Immunohistochemical detection results of TNF-α in rat skin. (**G**) Statistical graph of AIOD for IL-6 immunohistochemical detection. (**H**) Statistical graph of AIOD for TNF-α immunohistochemical detection. (**a**) Control group. (**b**) Model group. (**c**) GAg (positive control group). (**d**) G0 (blank matrix group, Matrix). (**e**) G3 (BBR@Matrix group). (**f**) G4 (AS@Matrix group). (**g**) G2 (BBR-AS@Matrix group). (**h**) G1-L (BBR-AS@Ce6@Matrix low-concentration group). (**i**) G1-H (BBR-AS@Ce6@Matrix high-concentration group). The results represent the mean ± SD of 6 independent experiments. *** *p* < 0.001 vs. Model group, ^△△^ *p* < 0.01, ^△△△^ *p* < 0.001 vs. Matrix group, ^##^ *p* < 0.01, ^###^ *p* < 0.001 vs. G2 group.

Immunohistochemical staining was performed to evaluate the protein expression levels of VEGF and CD31, two key markers of angiogenesis, with quantitative analysis of MOD values presented in Figure 10A–D. As shown in Figure 10A,C, the Model group exhibited markedly lower VEGF and CD31 immunostaining intensity compared with the Control group, and the corresponding MOD values were significantly reduced (*p* < 0.01; Figure 10B,D). This substantial decrease in angiogenic marker expression is consistent with impaired vascularization, likely resulting from the infectious injury-induced disruption of the local microvascular environment. Following treatment, both G1-H and G1-L groups showed notably stronger positive staining for VEGF and CD31 relative to the Model group (Figure 10A,C). Quantitatively, the MOD values in these two groups were significantly elevated (*p* < 0.01; Figure 10B,D), and reached levels comparable to those of the Control group, with no statistically significant difference detected between the G1-H/G1-L groups and the Control group (*p* > 0.05). This restoration of angiogenic marker expression suggests effective revascularization and tissue repair promoted by the treatment. To distinguish the therapeutic contribution of the active component from that of the delivery matrix, we compared the G1-H and G1-L groups with the G0 and G2 groups. As clearly shown in Figure 10B,D, the MOD values of both G1-treated groups were significantly higher than those of the G0 and G2 groups (*p* < 0.01), whereas no significant difference was observed between the G0 and G2 groups (*p* > 0.05). These quantitative comparisons confirm that the observed angiogenic response was specifically attributable to the Ce6 nanoparticle-loaded hydrogel, rather than to nonspecific effects of the matrix alone. Collectively, these findings provide strong evidence that Ce6 nanoparticle loading effectively promotes wound healing by enhancing local angiogenesis.

Immunohistochemical staining for the pro-inflammatory cytokines TNF-α and IL-6 was performed on skin tissue sections, with representative images shown in Figure 10E,G and corresponding MOD quantification presented in Figure 10F,H. As clearly shown in Figure 10F,H, the Model group exhibited a marked increase in MOD values for both TNF-α and IL-6 compared with the Control group (*p* < 0.01). This significant elevation in inflammatory cytokine expression confirms that the skin infectious injury successfully induced a robust local inflammatory response. Following treatment, the G1-H and G1-L groups showed substantially reduced immunostaining intensity for both cytokines relative to the Model group (Figure 10E,G). Quantitatively, the MOD values in these two treatment groups were significantly lower than those of the Model group (*p* < 0.01 for both; Figure 10F,H), indicating that the G1-based formulations effectively suppressed the infection-induced inflammatory cascade. To further verify that the observed anti-inflammatory effect was specifically attributable to the active components rather than to nonspecific actions of the delivery system, we compared the G1-H and G1-L groups with the G0 (blank matrix) and G2 groups. As shown in Figure 10F,H, both G1-treated groups exhibited significantly lower MOD values for TNF-α and IL-6 than did the G0 and G2 groups (*p* < 0.01), while no significant difference was detected between the G0 and G2 groups (*p* > 0.05). These quantitative comparisons confirm that the BBR-AS cocrystal and Ce6 nanoparticle-loaded hydrogel exerted a synergistic anti-inflammatory effect that was superior to that of the blank matrix or the single-component formulation alone, highlighting the therapeutic advantage of the combined active-ingredient delivery system.

### 2.12. Discussion

This study targeted two critical bottlenecks in infected wound therapy: the inefficient delivery of poorly soluble natural drugs and photosensitizers, and the lack of verifiable synergistic therapeutic strategies for refractory wound infection. We systematically constructed a chitosan-based nano-delivery platform and a composite responsive hydrogel system, supplemented with comprehensive physicochemical characterization, microbiological evaluation, and in vivo wound healing verification. Different from the purely descriptive result interpretation in conventional studies, this section further dissects the independent functional contribution of each core component (BBR-AS cocrystal, Ce6 photosensitizer, chitosan, and composite hydrogel matrix) and clarifies the intrinsic synergistic mechanism among components, establishing a solid mechanistic correlation between material structure, component function, and final therapeutic efficacy.

Photodynamic therapy (PDT) has emerged as a minimally invasive, highly targeted, and low-toxicity therapeutic modality for infectious diseases, with negligible risk of inducing bacterial drug resistance. The Ce6 photosensitizer possesses superior singlet oxygen generation capacity and photodynamic antibacterial activity compared with traditional photosensitizers, yet its inherent poor water solubility, aqueous aggregation behavior, and low cellular bioavailability severely restrict its standalone clinical application [18]. To address this defect, we adopted biocompatible chitosan as a nano-carrier matrix. Chitosan is characterized by abundant amino and hydroxyl groups, excellent hydrophilicity, and good tissue compatibility, and can form stable spherical nanoparticles via electrostatic interaction and ionic crosslinking with STPP [19]. In this study, a Ce6@CS nano-delivery system was prepared using this approach. By leveraging its hydrophilic shell–hydrophobic core structure, the system efficiently encapsulated and protected Ce6, thereby improving its solubility and stability. Through process optimization, the nanoparticles achieved an encapsulation efficiency of 84.59 ± 0.003% and a drug loading of 17.75 ± 0.522%, with uniform particle size and good dispersibility. The established UV–Vis spectrophotometric method for Ce6 quantification complied with pharmacopoeial requirements. Upon light irradiation, the nanoparticles efficiently generated singlet oxygen, demonstrating excellent photodynamic activity, thus successfully addressing the delivery bottleneck of Ce6.

In the fabricated Ce6@CS nano-delivery system, chitosan acts as a protective and delivery carrier with core functional advantages: the hydrophilic CS shell endows the nanoparticles with excellent water dispersibility and serum stability, while the hydrophobic inner core efficiently encapsulates Ce6 via hydrophobic interaction, fundamentally solving the solubility and aggregation problems of free Ce6. Process optimization achieved a high Ce6 encapsulation efficiency (84.59 ± 0.003%) and drug loading capacity (17.75 ± 0.522%), with uniform particle size distribution and favorable monodispersity. The validated pharmacopoeia-compliant UV–Vis quantification method ensured accurate drug release detection. Functionally, the CS carrier does not interfere with the photodynamic properties of Ce6; instead, it improves the cellular internalization of Ce6, enabling the nanoparticles to rapidly generate abundant singlet oxygen under light irradiation and retain robust photodynamic activity. This confirms the independent delivery and functional enhancement effect of chitosan carrier on Ce6, thoroughly breaking through the delivery bottleneck of the photosensitizer.

Berberine (BBR) and asiaticoside (AS) are two classic natural active ingredients with complementary pharmacological functions: BBR exerts broad-spectrum chemical antibacterial and anti-inflammatory effects, while AS dominates in promoting fibroblast proliferation, collagen deposition and wound tissue remodeling. However, the poor water solubility and burst release behavior of free BBR and AS greatly weaken their dual antibacterial and pro-healing efficacy, failing to achieve coordinated therapeutic effects. To solve this problem, we first constructed a BBR-AS cocrystal to optimize the physicochemical properties of the dual drugs. Different from physical mixture, the BBR-AS cocrystal forms a stable intermolecular hydrogen bond network, which significantly improves the aqueous solubility and dispersion uniformity of BBR and AS, and realizes preliminary modulation of drug release behavior, laying a material foundation for subsequent synergistic therapy. On this basis, we fabricated a pH-responsive three-dimensional network hydrogel matrix via Schiff base reaction between chitosan and oxidized sodium alginate. The hydrogel matrix serves as a macroscopic sustained-release platform and wound repair scaffold: its porous crosslinked network structure can stably load BBR-AS cocrystals and Ce6@CS nanoparticles, avoid rapid drug loss at the wound site, and achieve long-term sustained drug release matching the wound healing cycle. Moreover, the pH-responsive Schiff base bond can respond to the acidic microenvironment of infected wounds, trigger targeted drug release at the lesion site, and reduce systemic drug leakage.

To integrate the advantages of cocrystal drugs and nano-photosensitizers, we introduced Ce6@CS nanoparticles into the BBR-AS loaded hydrogel to construct a dual-delivery composite system (BBR-AS@Ce6@Matrix). Herein, Ce6@CS nanoparticles play a critical auxiliary regulatory role in hydrogel drug delivery: the uniformly dispersed nanoparticles can refine the internal pore structure of the hydrogel, form microchannels for medium penetration, and reduce the intermolecular aggregation of BBR-AS cocrystals. This structural optimization effectively promotes the diffusion and dissociation of loaded drugs, significantly improving the cumulative release efficiency of BBR and AS compared with the pure drug-loaded hydrogel. In vitro cellular and reactive oxygen species (ROS) verification further confirmed that the photodynamic function of the composite system is completely derived from Ce6, and the CS carrier and hydrogel matrix only act as structural supports and delivery media without quenching ROS activity. This realizes the effective coupling of nano-delivery optimization and macroscopic hydrogel sustained-release function, forming a complete functional verification chain from component design to system integration.

Infected wounds are clinically characterized by persistent bacterial infection, excessive inflammatory response, and blocked tissue regeneration, while traditional antibiotic treatment easily induces bacterial resistance and delayed wound healing [20]. In this study, the composite hydrogel was subjected to in vitro antibacterial evaluation and in vivo wound healing assessment. The in vitro results demonstrated excellent antibacterial activity against *Escherichia coli* and *Staphylococcus aureus*. Notably, the inhibitory effect against *S. aureus* appeared to be at least as effective as that of a clinically used silver ion gel, suggesting potential for further comparative evaluation. The excellent in vitro and in vivo therapeutic performance of our composite hydrogel originates from the verifiable multi-component synergistic mechanism, rather than simple superposition of single functions, which clarifies the connotation of “multifunctional and synergistic effects” proposed in this study. First, the BBR-AS cocrystal provides chemical antibacterial and pro-healing baseline effects: BBR damages bacterial cell membrane integrity and inhibits bacterial metabolic activity, while AS regulates wound microenvironment and promotes cell migration, realizing the coordination of antibacterial and tissue repair functions at the drug level. Second, Ce6-mediated PDT provides physical antibacterial enhancement effect: under light irradiation, Ce6 generates high levels of ROS, which irreversibly oxidizes bacterial proteins, lipids and nucleic acids, killing drug-resistant bacteria that are insensitive to chemical drugs and compensating for the insufficient antibacterial persistence of single chemical therapy. Third, chitosan exerts auxiliary synergistic antibacterial and bioadhesive effects: the positively charged amino groups on chitosan can adsorb negatively charged bacterial cell walls, cause bacterial cell rupture and death, and its good tissue adhesion enables the hydrogel to fit closely with irregular wound surfaces, prolonging the residence time of drugs and photosensitizers at the lesion site. The three functional components act through independent and complementary pathways, forming a dual chemical-physical antibacterial system with high efficiency, long duration and low drug resistance risk.

In vitro antibacterial experiments verified that the composite hydrogel exhibits excellent inhibitory activity against both *Escherichia coli* (Gram-negative) and *Staphylococcus aureus* (Gram-positive), with its antibacterial effect against S. aureus being comparable to that of clinical silver ion gel, indicating good potential for clinical application. Notably, the hydrogel showed stronger activity against Gram-positive bacteria, which may be attributed to structural differences in the bacterial cell wall: Gram-positive bacteria lack an outer membrane, making them more susceptible to ROS generated by PDT. The hydrogel matrix, particularly due to the presence of chitosan, may influence ROS generation and diffusion. While the positive charge of chitosan can capture negatively charged bacteria and enhance local ROS concentration, the hydrogel network may also restrict the diffusion distance of short-lived ROS, representing an inherent limitation of PDT. Additionally, oxygen dependency remains a key constraint of PDT, as its efficacy is significantly reduced under hypoxic conditions commonly found in infected or necrotic wound tissues.

The hydrogel also demonstrated favorable biocompatibility, promoting the proliferation and migration of wound repair-related cells. In a rat infected wound model, the synergistic effect of BBR-AS cocrystal-based chemotherapy and Ce6-mediated PDT rapidly eliminated wound infection, inhibited excessive inflammatory infiltration, and promoted granulation tissue hyperplasia and collagen fiber remodeling. Under light irradiation, therapeutic efficacy was significantly enhanced, confirming a true synergistic interaction among the components. From an integrated biological perspective, the early antibacterial action reduces pathogen-associated inflammatory stimuli, thereby suppressing the overexpression of pro-inflammatory cytokines (e.g., IL-1β, TNF-α). This creates a favorable microenvironment for subsequent angiogenesis, as supported by increased CD31 expression, and for fibroblast activation and collagen remodeling, ultimately achieving coordinated regulation of antibacterial, anti-inflammatory, and pro-healing functions.

Despite these promising results, several limitations of the system should be critically acknowledged. The efficacy of PDT remains highly dependent on light delivery and tissue oxygen levels, posing challenges for treating deep or hypoxic infections. Moreover, the match between hydrogel degradation rate and tissue repair rate requires further optimization. Regarding clinical translation, key challenges include ensuring the safety and long-term stability of the photosensitizer, the feasibility and standardization of light irradiation devices in clinical settings, and the scalability of manufacturing and storage. Future studies should systematically address these issues to facilitate the clinical application of this system.

## 3. Conclusions

This study constructs an innovative integrated therapeutic system based on BBR-AS cocrystals, chitosan nano-carriers and photodynamic therapy, which systematically solves the delivery defects of poorly soluble natural drugs and photosensitizers. By clarifying the unique functional orientation of each core component and their complementary synergistic pathways, this work provides clear experimental and mechanistic support for infected wound treatment. Nevertheless, this study still has certain limitations: the current mechanism verification is limited to phenotypic and functional levels, and the molecular regulatory mechanism of component synergism has not been elucidated; the biosafety evaluation is restricted to short-term in vitro and in vivo detection; and the therapeutic effect is only verified in conventional acute infected wounds, without exploring refractory wounds such as diabetic ulcers and burn wounds. Future research will focus on exploring the molecular signaling pathways mediated by the synergistic system, conducting long-term in vivo biosafety assessment, optimizing the component ratio and preparation process of the hydrogel system, and expanding its application scenarios, so as to further promote the clinical translation of this multifunctional delivery system and provide a feasible strategy for the application of chitosan-based polysaccharide delivery carriers in poorly soluble drug therapy.

## 4. Materials and Methods

### 4.1. Materials

Berberine (Lot No.: AB0530) and Asiaticoside (Lot No.: AB1474) were purchased from Chengdu Aifa Biotechnology Co., Ltd. (Chengdu, China); low molecular weight chitosan (Lot No.: C17042800, molecular weight = 200 kDa) with a deacetylation degree of 85.0% (5 to 20 mPa·s, 0.5% in 0.5% acetic acid at 20 °C), chlorine e6 (Lot No.: C17005891), and sodium alginate (Lot No.: S749909, M/G ratio = 1:2, molecular weight: 140–150 K, viscosity (1% solution): 850–1000 mPa·s) were purchased from Shanghai Macklin Biochemical Technology Co., Ltd. (Shanghai, China); sodium tripolyphosphate (Lot No.: RH546180) and sodium periodate (Lot No.: RH705067) were purchased from Guangzhou Ron Biological Technology Co., Ltd. (Guangzhou, China); N,N-dimethylformamide (Lot No.: O2EXIRRD) was purchased from Anhui Zesheng Technology Co., Ltd. (Hefei, China); Fetal bovine serum (Lot No.: 10099-141C) was purchased from Gibco (Waltham, MA, USA); high-glucose DMEM basal medium (Lot No.: L211212) was purchased from Shanghai Yuanpei Biotechnology Co., Ltd. (Shanghai, China).; nutrient broth medium (Lot No.: not retrieved) was purchased from Beijing Beina Chuanglian Biotechnology Research Institute (Beijing, China); nutrient agar (Lot No.: 20241120) was purchased from Qingdao High-Tech Industrial Haibo Biotechnology Co., Ltd. (Qingdao, China); VEGF antibody (Lot No.: MP00561-4), CD31 antibody (Lot No.: MP01441-2), IL-6 antibody (Lot No.: MP00756-4), and TNF-α antibody (Lot No.: MP00682-1) were purchased from Wuhan Sanying Biotechnology Co., Ltd. (Wuhan, China)

#### 4.1.1. Bacteria and Cells

*Staphylococcus aureus* (186335) and *Escherichia coli* (336902) were purchased from Beijing Beina Chuanglian Biotechnology Research Institute; Mouse fibroblast cell line (L929, C5131) and human keratinocyte cell line (HaCaT, C5070) were purchased from Zhejiang Baidi Biotechnology Co., Ltd. (Hangzhou, China). To ensure consistent cytotoxic response in accordance with ISO 10993-5 [21], all experiments were performed using L929 cells within passage 20. For keratinocyte differentiation and skin inflammation assays, HaCaT cells below passage 30 were utilized to maintain normal epidermal differentiation phenotype.

#### 4.1.2. Experimental Animals

A total of 81 healthy SPF-grade SD rats, half male and half female, aged 6 weeks and weighing 180–220 g, were provided by Hunan SJA Laboratory Animal Co., Ltd. (Changsha, China). All SD rats were housed in cages (3 per cage) in an SPF-grade animal facility under a 12 h light/12 h dark cycle, with the ambient temperature controlled at 22 ± 2 °C and relative humidity maintained at 50–60%. The animals had free access to standard maintenance diet and sterilized water. All experiments were conducted after one week of acclimatization feeding. All animal studies were approved by the Ethics Committee of Zunyi Medical University (Permit No. ZMU21-2503-287) and performed in accordance with the Laboratory Animal Welfare and Ethics Committee of China.

### 4.2. Methods

#### 4.2.1. Preparation and Purification of BBR-AS Cocrystal

Berberine (BBR) and asiaticoside (AS) were weighed in a 1:1 molar ratio, mixed, and dissolved in an appropriate amount of anhydrous ethanol solution to form a homogeneous liquid. The mixture was slowly stirred at room temperature (25 °C) for 48 h, then allowed to stand and naturally evaporate to dryness at 25 °C for 12 h. The resulting BBR-AS cocrystal was collected and its melting point was determined.

#### 4.2.2. Characterization of BBR-AS Cocrystal

(1) SEM Detection

Scanning electron microscopy (SEM) was used to observe the surface morphology of BBR, AS, BBR-AS physical mixture, and BBR-AS cocrystal. The specific experimental procedure was as follows: 5 mg of each sample was accurately weighed and uniformly dispersed onto a sample stage coated with conductive adhesive. A rubber bulb was used to gently blow off any loosely attached powder. Nitrogen was used as a protective gas. A gold film with a thickness of less than 1 nm was sputter-coated onto the sample surface using an ion sputter coater (sputtering current: 10 mA, duration: 30 s). The accelerating voltage was set to 3 kV or 5 kV, and an appropriate magnification was selected based on the sample characteristics to obtain clear, high-resolution surface morphology images.

(2) X-ray Diffraction (XRD) Analysis

XRD was used to characterize AS, BBR, the BBR-AS physical mixture at a 1:1 stoichiometric ratio, and the BBR-AS cocrystal. The experimental conditions were as follows: 50 mg of each sample was uniformly packed into the sample holder. A 2θ scanning mode was adopted, with a tube voltage of 40 kV, a tube current of 40 mA, a divergence slit (DS) of 0.6 mm, and a scanning speed of 6°/min. Cu target (wavelength: 1.5406 Å) and Co target (wavelength: 1.79026 Å) were used as X-ray sources to obtain high-resolution diffraction patterns. The characteristic peaks of the diffraction patterns of each sample were compared and analyzed to evaluate the formation of the BBR-AS cocrystal and the resulting changes in its crystal structure.

(3) Differential Scanning Calorimetry (DSC) Analysis

The thermal behavior of BBR, AS, physical mixture, and BBR-AS cocrystal samples was analyzed using a DSC thermal analyzer. The specific experimental conditions were as follows: approximately 15 mg of each sample was placed into a pure aluminum crucible. Nitrogen was used as a protective gas at a flow rate of 20 mL/min. The temperature ramp ranged from 30 °C to 500 °C at a heating rate of 20 °C/min.

(4) Fourier Transform Infrared Spectroscopy (FTIR) Analysis

The BBR, AS, physical mixture, and BBR-AS cocrystal samples were systematically characterized using FTIR. The specific experimental steps were as follows: First, an appropriate amount of each sample was mixed with dried potassium bromide (KBr) at a ratio of 1:100, ground evenly, and pressed into a transparent thin pellet. Second, the pellet was placed into the FTIR sample compartment. The number of scans was set to 32 to improve the signal-to-noise ratio, the resolution was set to 4 cm^−1^ to ensure spectral clarity, and the wavelength range was 4000–400 cm^−1^ to cover the characteristic vibrational frequencies of the samples. By comparing and analyzing the spectra of each sample, the molecular interactions between BBR and AS could be identified, and the formation of the BBR-AS cocrystal along with its structural characteristics could be verified.

(5) Drug Release Experiment

Excess amounts of BBR, AS active pharmaceutical ingredients, and BBR-AS cocrystals were weighed and placed into dissolution vessels containing 50 mL of 0.005% Tween-80 aqueous solution. The apparatus was set to a rotation speed of 250 r/min and a temperature of (37.0 ± 0.5) °C. Timing was started from the moment the drugs came into contact with the dissolution medium. Samples of 150 μL were taken at 6, 12, 18, 24, 30, 45, 60, 120, and 240 min (while an equal volume of fresh dissolution medium at the same temperature was simultaneously replenished), filtered, and 20 μL of the subsequent filtrate was taken for determination by HPLC.

#### 4.2.3. Preparation of Ce6-Loaded Chitosan Nanoparticles (Ce6@CS)

Chitosan nanoparticles were prepared via the nonsolvent-assisted counterion complexation crosslinking method. Briefly, a 1.3 mg/mL chitosan (CS) aqueous solution and a 1 mg/mL sodium tripolyphosphate (STPP) aqueous solution were prepared. A mixture of 8 mL of anhydrous ethanol and 2 mL of CS aqueous solution was stirred at 500 rpm, and 338 μL of STPP solution was slowly added dropwise to achieve a CS:STPP mass ratio of 7.7:1. After reaction at room temperature for 1 h, the nanoparticles were purified by ultrafiltration (100 kDa, 2000 rpm, 15 min, three washes with pure water) and freeze-dried to obtain blank chitosan nanoparticles.

For the preparation of Ce6-loaded chitosan nanoparticles (Ce6@CS), the same procedure was followed, except that an appropriate amount of Ce6 (1 mg/mL in anhydrous ethanol, 0.78 mL) was dissolved in the CS/ethanol mixture prior to the addition of STPP solution, corresponding to a CS: Ce6 mass ratio of 1:0.3. The subsequent crosslinking, purification, and freeze-drying steps were identical to those described above.

#### 4.2.4. In Vitro Singlet Oxygen Detection of Ce6@CS

1,3-Diphenylisobenzofuran (DPBF) exhibits high specificity toward reactive oxygen species (ROS), resulting in a decrease in its ultraviolet absorbance. Therefore, DPBF was used as a ROS probe to evaluate the photodynamic properties of Ce6@CS and detect the singlet oxygen generation of Ce6@CS solution and Ce6 solution. Briefly, 6 mg of DPBF was dissolved in 2 mL of N, N-dimethylformamide (DMF). Then, 15 μL of DPBF solution was added to pure water, Ce6 solution, and Ce6@CS solution with identical Ce6 content, respectively, with a total volume of 2 mL. Under irradiation with a 660 nm laser at a power density of 100 mW/cm^2^, the absorbance of DPBF at its maximum ultraviolet absorption wavelength was measured every 60 s.

#### 4.2.5. Preparation of Hydrogels Loaded with BBR-AS Cocrystals and Ce6@CS Nanoparticles

10 g of sodium alginate (SA) was dispersed in 100 mL of anhydrous ethanol and stirred uniformly. 9.89 g of NaIO_4_ (equimolar to SA) was dissolved in 100 mL of pure water to form a homogeneous aqueous solution. The two solutions were then mixed and reacted in the dark at room temperature for 6 h. Subsequently, ethylene glycol equimolar to NaIO_4_ was added to quench the reaction for 0.5 h. The reaction mixture was poured into a large amount of anhydrous ethanol for precipitation. After discarding the supernatant, the lower precipitate was transferred into a dialysis bag (molecular weight cutoff: 3500 Da) and dialyzed against pure water for 3 days, with water renewed every 8 h. Finally, the solution inside the dialysis bag was collected and freeze-dried to obtain purified oxidized sodium alginate (OSA). 0.25 g of chitosan (CS) was dissolved in 2.5 mL of 2% acetic acid solution and stirred at room temperature to prepare a 0.1 g/mL CS solution, which was then adjusted to approximately pH 7 with 0.2 mol/L NaOH solution. 2 g of OSA was dissolved in 20 mL of pure water and stirred at room temperature to prepare a 0.1 g/mL OSA solution. Separately, 10 mg of BBR-AS cocrystal was dissolved in 1 mL of pure water (or Ce6@CS aqueous solution) to prepare a 10 mg/mL BBR-AS solution (or BBR-AS solution containing Ce6@CS). 1 mL of pure water (BBR-AS solution or Ce6@CS-containing BBR-AS solution) was added to the CS solution and mixed uniformly. Under magnetic stirring at 1000 rpm, 800 μL of 0.1 g/mL OSA solution was slowly added dropwise. After stirring uniformly and standing at room temperature for 4 h, blank hydrogel (matrix hydrogel, denoted G0), BBR-AS-loaded hydrogel (BBR-AS@Matrix, denoted G2), and BBR-AS and Ce6 co-loaded hydrogel (BBR-AS@Ce6@Matrix, denoted G1) were obtained, respectively.

BBR-loaded hydrogel (BBR@Matrix, denoted G3) and AS-loaded hydrogel (AS@Matrix, denoted G4) were prepared using the same method. Among G1 samples, those with a BBR-AS concentration of 10 mg/mL were designated G1-H, and those with 5 mg/mL were designated G1-L. SEM was employed to characterize the Matrix hydrogel and BBR-AS@Ce6@Matrix hydrogel.

#### 4.2.6. Characterization of Hydrogels Loaded with BBR-AS Cocrystals and Ce6@CS Nanoparticles

##### Rheological Properties of Hydrogels

Rheological tests were performed using a rotational rheometer in oscillatory mode with a 25 mm diameter rotor. Amplitude sweep was carried out at a frequency of 1 Hz over a strain range of 0.1–100% to determine the linear viscoelastic region (LVR) of the hydrogels. Within the LVR, frequency sweep was conducted at a fixed strain of 1% over a frequency range of 0.1–100 rad/s to measure the storage modulus (G’) and loss modulus (G’’) of the hydrogels. At a fixed frequency of 1 Hz, three consecutive cycles of alternating low strain (1%) and high strain (300%) oscillatory amplitude sweep tests were performed.

##### Physicochemical Characterization of Hydrogels

(1) Water Content

Freshly prepared Matrix hydrogel and BBR-AS@Ce6@Matrix hydrogel were weighed to record their initial mass (m_0_), then dried to constant weight at 37 °C in a forced airdrying oven, and the final mass (m_d_) was measured. The water content was calculated using Equation:

Water Content (%) = (m_0_ − m_d_)/m_0_ × 100%

(2) Water Retention Rate

Freshly prepared Matrix hydrogel and BBR-AS@Ce6@Matrix hydrogel were weighed to record their initial mass (m_0_) and placed at room temperature. The mass (m_t) was measured at 2, 4, 6, 8, 12, and 24 h, respectively. The water retention rate was calculated using Equation:

Water Retention Rate (%) = m_t_/m_0_ × 100%

(3) Swelling Behavior

Three parallel samples of each freshly formed hydrogel (Matrix and BBR-AS@Ce6@Matrix) were prepared and their initial mass (m_0_) was recorded. The weighed hydrogels were immersed in 20 mL of PBS solution at room temperature. At predetermined time intervals (0.5, 1, 2, 4, 6, 8, 12, and 48 h), the hydrogel samples were taken out, blotted with filter paper to remove surface water, and weighed (m_t_). The swelling ratio was calculated using the following Equationuntil the hydrogel weight remained constant and reached equilibrium swelling:

Swelling rate (%) = (m_t_ − m_0_)/m_0_ × 100%

(4) Degradation Behavior

Three parallel samples of each freshly formed hydrogel (Matrix and BBR-AS@Ce6@Matrix) were prepared and their initial mass (m_0_) was recorded. The weighed hydrogels were immersed in 20 mL of PBS solutions (pH 5.5, pH 6.8, and pH 7.4) containing 1.5 mg/mL lysozyme, and incubated in a constant temperature water bath shaker at 37 °C with a shaking speed of 50 rpm. At 0.5, 1, 2, 4, 6, 8, 12, and 48 h, the hydrogel samples were removed, blotted with filter paper to remove the release medium on the surface, and immediately weighed accurately (m_n_). After weighing, the samples were returned to the corresponding PBS solution for continuous incubation. The lysozyme-containing PBS solution was renewed every 12 h. The cumulative degradation ratio at each time point was calculated in triplicate using Equation:

Degradation rate (%) = (m_0_ − m_n_)/m_0_ × 100%

#### 4.2.7. ROS Detection

Detection of intracellular ROS levels in Ce6@ CSNPs-treated L929 cells following 660 nm laser irradiation using the DCFH-DA fluorescent probe method. L929 cells were seeded and cultured, then divided into a blank CS NPs group and a Ce6@CS NPs group. Cells in both groups were exposed to 660 nm laser irradiation for 15, 30, and 45 min. After irradiation, cells were incubated with DCFH-DA probe diluted in serum-free medium for 20 min at 37 °C in the dark. Following probe loading, cells were washed with PBS to remove free probe, and fluorescence signals were immediately observed under a fluorescence microscope. The fluorescence intensity is positively correlated with intracellular ROS levels. Comparative analysis of fluorescence values among groups was used to evaluate the ROS levels generated by Ce6@CSNPs upon 660 nm laser excitation.

#### 4.2.8. In Vitro Antibacterial Test

In vitro antibacterial experiments were conducted using Gram-positive *Staphylococcus aureus* and Gram-negative *Escherichia coli*. The antibacterial performance of the hydrogels against *Escherichia coli* and *Staphylococcus aureus* was evaluated by the plate colony counting method and the inhibition zone method [22]. The detailed procedures were as follows:

(1) Plate colony counting method: The antibacterial activities of the matrix hydrogel (Matrix, G0), BBR-AS@Ce6@Matrix (G1), BBR-AS@Matrix (G2), and silver ion disinfectant gel (GAg, used as the positive control) groups were compared. A bacterial suspension (10^5^ CFU/mL, 500 μL) was added to an equal volume of each hydrogel sample. The G1 group was irradiated with a light source (wavelength 660 nm, 100 W/cm^2^) for 10 min. Subsequently, the hydrogels were co-incubated with the bacterial solution at 37 °C for 2 h. The bacteria were then resuspended in an equal volume of PBS. For the control group, the bacterial suspension was dispersed in an equal volume of PBS. After thorough mixing, 100 μL of the bacterial suspension from each group was evenly spread onto agar plates. Following incubation at 37 °C for 24 h, the number of colonies on the agar plates was observed.
(2) Oxford cup method: Bacterial suspensions of *E. coli* and *S. aureus* (1 × 10^5^ CFU/mL) were spread onto agar plates. After the plates were air-dried, Oxford cups were placed vertically on the surface, and each group of hydrogel (G0, G1, G2, GAg) was added into the cups, with GAg serving as the positive control. The plates were incubated at 37 °C for 12 h, after which the diameters of the inhibition zones were measured.

The morphology of normally growing bacteria (*E. coli* and *S. aureus*) and bacteria treated with G2 or G1 plus light irradiation was characterized by scanning electron microscopy (SEM) and transmission electron microscopy (TEM) to determine the mode of bacterial death.

#### 4.2.9. Cell Experiments

##### Cytotoxicity Evaluation of Hydrogels

L929 and HaCaT cells are frequently used to evaluate the in vitro biocompatibility of biomaterials. Therefore, the cytotoxicity of the hydrogels was evaluated using the CCK-8 assay in L929 and HaCaT cells. The experimental groups included the Control group, the matrix hydrogel (Matrix, G0), BBR-AS@Ce6@Matrix (G1), BBR@Matrix (G3), and AS@Matrix (G4). First, hydrogel extracts were prepared by soaking the hydrogels in DMEM at 4 °C for 24 h. Five concentration gradients (0.2, 0.1, 0.05, 0.025, and 0.0125 g/mL) were set for each group. A blank control group and a solvent control group were also included. HaCaT cells and L929 cells were seeded into 96-well plates at a density of 5 × 10^3^ cells per well. After 24 h of adherent growth, the culture medium was discarded, and 100 μL of the hydrogel extract was added to each well, followed by incubation for 24 h. Subsequently, the hydrogel extract was discarded, and 100 μL of culture medium containing 10% CCK-8 reagent was added, followed by incubation for 1.5 h. The absorbance (OD) value was measured at 450 nm using a microplate reader.

##### Cell Scratch Assay of Hydrogels

Well-grown L929 cells and HaCaT cells were respectively seeded into 6-well plates at a density of 1 × 10^6^ cells per well. When the cell density reached approximately 80–90%, scratches were made using a 200 μL pipette tip, and the cells were washed three times with PBS. Based on the cytotoxicity results, hydrogel extracts at two concentration gradients prepared in cell maintenance medium were added, and a control group was also set up. The plates were then incubated in a cell culture incubator. Cell migration was observed and photographed at 0, 24, 36, and 48 h. The scratch areas were quantified using ImageJ software1.7.9, and the cell migration rate was calculated according to the following formula:

Migration rate (%) = (Scratch area at 0 h–Scratch area at 24 h)/Scratch area at 0 h × 100%

#### 4.2.10. Animal Experiments

(1) Animal Model Establishment and Drug Administration

To evaluate the application potential of the BBR-AS@Ce6@Matrix hydrogel, 81 healthy SPF-grade SD rats were acclimatized for three days and then randomly divided into 9 groups, including a negative control group with untreated skin defects (Control group), a Model group, a positive control drug group (silver ion disinfectant gel, GAg), a high-dose BBR-AS@Ce6@Matrix + 660 nm laser irradiation group (G1-H), a low-dose BBR-AS@Ce6@Matrix + 660 nm laser irradiation group (G1-L), a BBR-AS@Matrix group (G2), a BBR@Matrix group (G3, containing an amount of BBR equivalent to that in G1-H), an AS@Matrix group (G4, containing an amount of AS equivalent to that in G1-H), and a matrix hydrogel group (G0). To establish E. coli and S. aureus infected wound models, the rats were anesthetized by intraperitoneal injection of tribromoethanol (2.5%) before surgery. The upper back of each rat was shaved and disinfected, and a circular full-thickness skin wound (8 mm in diameter) was created in the shaved dorsal region using a skin punch. Except for the Control group, each group was inoculated with 100 μL of E. coli and S. aureus suspension (1 × 10^6^ CFU/mL) to establish a full-thickness skin defect infection model. The day of surgery was designated as Day 0, and the day after surgery was considered the first day of treatment. Different groups of hydrogels were applied to the infected wound sites, with treatment administered once daily, and the healing effects of the hydrogels were compared among the groups. Wound areas were photographed on Days 3, 7, and 12 of treatment. The wound healing rate was used to calculate the CI index, and the therapeutic effect of the BBR-AS@Ce6@Matrix hydrogel on infected wounds was evaluated.

(2) H&E Staining

Fresh skin tissues were fixed in 4% paraformaldehyde solution for 24 h to preserve tissue structure and antigen integrity. The tissues were then rinsed with PBS buffer to remove residual paraformaldehyde. After dehydration with alcohol, the tissues were immersed in xylene until semi-transparent, embedded in paraffin, and sectioned. Finally, H&E staining was performed, followed by dehydration and sealing, and the skin pathological morphology was observed.

(3) Masson Staining

The pretreatment, embedding, and sectioning procedures were the same as those for H&E staining. Masson staining was then performed, followed by dehydration and sealing. The aggregation of hair follicles and collagen deposition at the skin wound site were observed and quantified.

(4) Immunohistochemical detection

The pretreatment, embedding, and sectioning procedures were the same as those for H&E staining. After deparaffinization, the sections were rehydrated with graded alcohols and washed with PBS. The slides were then immersed in citrate buffer solution, followed by washing with PBS. Subsequently, the sections were incubated with H_2_O_2_ solution for 10 min. After washing with PBS, the primary antibodies (IL-6/CD31/VEGF/TNF-α) diluted according to the manufacturer’s instructions were added dropwise, and the sections were incubated at 4 °C for 16 h. After three washes with PBS, the IgG antibody was added, and the sections were incubated at 37 °C for 30 min. Finally, DAB chromogenic reaction was performed. The chromogenic reaction was stopped immediately when a brown positive reaction was observed in the cytoplasm under the microscope. The sections were then observed and counted under a microscope.

#### 4.2.11. Statistical Analysis

Data are expressed as mean ± SEM. Group comparisons were primarily performed using one-way ANOVA followed by Tukey’s post hoc test. Histopathological semi-quantitative lesion scores were analyzed using the Kruskal–Wallis test with Dunn’s multiple comparison procedure. *p* < 0.05 was considered statistically significant. All statistical analyses and graphs were generated using GraphPad Prism 9.0 (GraphPad Software, San Diego, CA, USA).

## Data Availability

All data of this study are included in the paper.

## Supplementary Materials

The following supporting information can be downloaded at: https://www.mdpi.com/article/10.3390/gels12070620/s1, Table S1. The melting point results of BBR, AS and BBR-AS cocrystal; Table S2. Solubility results of AS and BBR-AS cocrystal; Table S3. Physicochemical parameters of blank nanoparticles and Ce6-loaded nanoparticles; Table S4. Statistical table of hydrogel antibacterial zone diameters; Figure S1. Singlet oxygen generation capability detection of Ce6@CS NPs. (A) Photodegradation of DPBF only; (B) Photodegradation of Ce6 + DPBF; (C) Photodegradation of Ce6@CS + DPBF; (D) Relationship between photodegradation absorbance of DPBF and irradiation time in DPBF (control group), Ce6, and Ce6@CS.
