# Supplementary material for: Study on the Preparation of a Photo-Responsive Hydrogel Loaded with Berberine–Asiaticoside Cocrystal and Its Therapeutic Effect on Infected Wounds
Source: Gels. 2026 Jul 9;12(7):620. doi: 10.3390/gels12070620 (PMC13409658; doi:10.3390/gels12070620)
Supplement: Supplementary file 1 [file gels-12-00620-s001.zip › gels-4323442-supplementary.pdf]

Supplemental Material

Study on the preparation of a photo-responsive hydrogel loaded with Berberine–Asiaticoside cocrystal and its therapeutic effect on infected wounds

Muxi Sui 1#, Jin Niu 2#, Shuwen Pang 1#, Shuang Zhao 1, Pingxi Zhou 1, Mengdi Zhao 1, Yongai Xiong 3 * and Jing Li 1 *

Table S1. The melting point results of BBR, AS and BBR-AS cocrystal

Sample	n	Melting range（℃）
BBR	3	201.7~203.1
AS	3	232.2~235.8
BBR-AS	3	227.8~229.4

Table S2. Solubility results of AS and BBR-AS cocrystal

Sample	n	solubility（μg/mL）
AS	3	61.18
BBR-AS	3	416.90

Table S3. Physicochemical parameters of blank nanoparticles and Ce6-loaded nanoparticles

Sample	Particle size（nm）	PDI	Encapsulation efficiency（%）	Drug Loading Efficiency （%）
CS	147.47±7.38	0.267±0.022	N/A	N/A
Ce6@CS	184.10±3.38	0.217±0.023	84.59±0.003	17.75±0.522

Table S4. Statistical table of hydrogel antibacterial zone diameters

Group	*E. coli* （mm）	*S. aureus*（mm）
G0	9.646±1.114	7.033±1.186
G2	14.581±1.691	15.328±0.263
G1	15.314±1.332	16.264±0.989
GAg	16.848±0.858	12.121±1.044

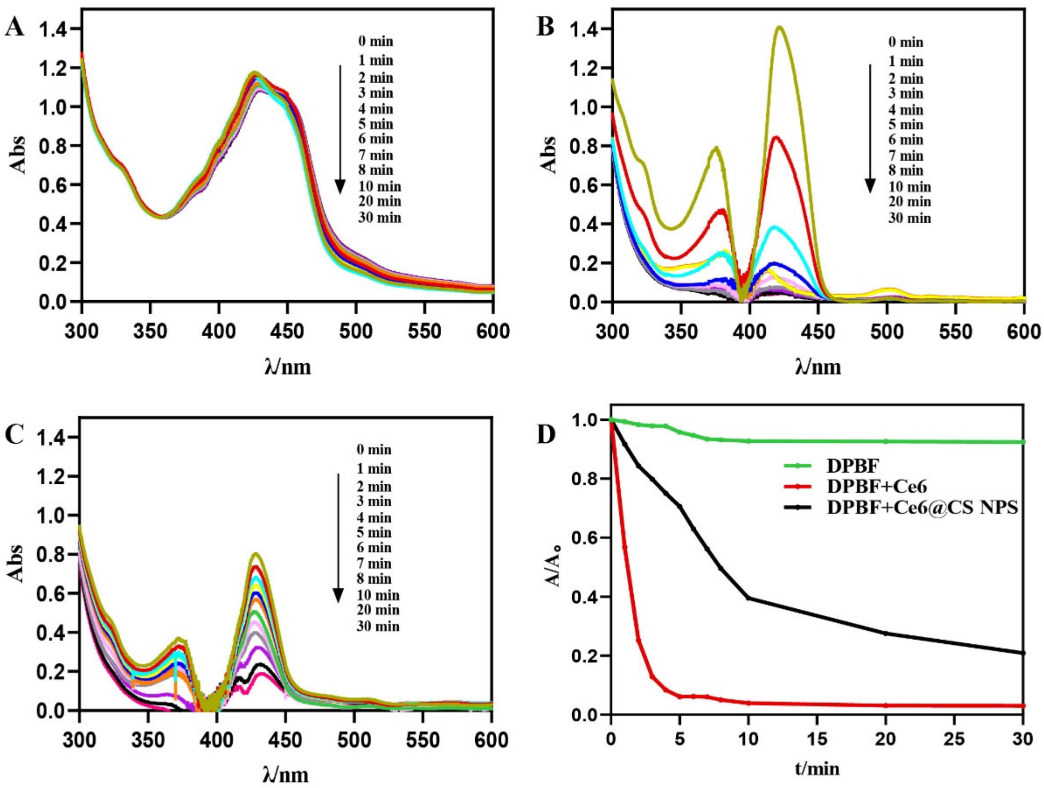

Figure S1. Singlet oxygen generation capability detection of Ce6@CS NPs. (**A**) Photodegradation of DPBF only; (**B**) Photodegradation of Ce6 + DPBF; (**C**) Photodegradation of Ce6@CS + DPBF; (**D**) Relationship between photodegradation absorbance of DPBF and irradiation time in DPBF (control group), Ce6, and Ce6@CS.
